# A Repeated Block Perturbation Subsampling for Large-Scale Longitudinal Data

**DOI:** 10.1007/s42519-026-00591-2

**Published:** 2026-06-05

**Authors:** Yujing Yao, Joseph H. Lee, Zhezhen Jin

**Affiliations:** 1 Gertrude H. Sergievsky Center, Taub Institute, and Department of Neurology, Columbia University, 630 W 168th St, New York, 10032 NY USA; 2 Department of Epidemiology, Mailman School of Public Health, Columbia University, 722 W 168th St, New York, 10032 NY USA; 3 Department of Biostatistics, Mailman School of Public Health, Columbia University, 722 W 168th St, New York, 10032 NY USA

**Keywords:** Mhealth, Perturbation, Subsampling

## Abstract

Large-scale longitudinal data are now commonly available due to technological advances and collaborative research efforts. One example is the use of mobile data collection app in healthcare studies (mHealth), which generates a large-scale longitudinal mHealth data. In this paper, we propose a repeated block perturbation subsampling algorithm for the analysis of large-scale longitudinal data based on generalized estimating equation. The proposed method simultaneously provides consistent point and variance estimators. We establish the asymptotic properties of the resulting subsampling estimators. We also examine the finite-sample performance of the proposed method with simulation studies and demonstrate its application using real mHealth data sets.

## Introduction

Billions of people worldwide now use smartphones with apps for a wide range of purposes [1]. Of the estimated 4.5 million apps available in the Google and Apple app stores, a subset of 300,000 may be regarded as bona fide mobile health (mHealth) apps, which is defined as medical and public health practices supported by mobile devices by World Health Organization [2, 3]. Millions of people worldwide are believed to have downloaded one or more mHealth apps to their mobile phone [4]. According to Statista Survey, the number of health app users in the United States exceeded 86 million in 2022 and continues to increase. The widespread adoption of mobile apps for data collection has resulted in large-scale longitudinal datasets, in which each subject contributes repeated measurements over time and the within-subject correlations among these repeated measures are non-negligible.

The generalized estimating equation (GEE) approach [5] is a semiparametric statistical method for the analysis of longitudinal data that fits marginal models. It is a population-level approach based on a quasi-likelihood function and provides the population-averaged parameter estimates, in contrast to mixed-effects models, which focuses on the individual-level interpretation [6, 7]. Moreover, GEE relaxes strict distributional assumptions and requires only correct specification of the first two moments of the response variable, along with an appropriate link function relating the covariates to the mean response [8]. However, in settings involving large-scale datasets such as mHealth data, the sample size of longitudinal data may exceed a single computer’s memory capacity, rendering direct computation of the GEE estimator infeasible. To analyze such massive datasets, one common solution is parallel or distributed computing [9–12]. An alternative approach is subsampling [13–22].

Kleiner et al. [23] proposed a scalable bootstrap method, known as the bag of little bootstraps (BLB), which combines features of both bootstrap and subsampling for massive datasets. They further extended BLB to time-series data through variants of the block bootstrap. Although extending these methods from the independent and identically distributed (i.i.d.) setting to dependent data is often convenient to implement, theoretical justification is generally nontrivial. Moreover, subsampling with or without replacement induces dependence among subsamples due to the underlying multinomial or multivariate hypergeometric distributions, respectively, resulting in negative correlations. These issues pose significant challenges for the analysis of large-scale dependent data. The key objective is therefore to mitigate the artificial dependence introduced by subsampling or bootstrapping while preserving, as much as possible, the intrinsic dependence structure of the original data.

As an alternative to the bootstrap, perturbation methods have been studied for statistical inference on parameters of interest based on a large collection of resulting perturbation estimators [24–26]. In this paper, we propose a new procedure, a repeated block perturbation subsampling method, that incorporates features of both Poisson subsampling and block perturbation for the analysis of large-scale longitudinal data. Elements in each subsample are selected via Poisson sampling [27], after which a perturbation is applied at the subject level to approximate the full-data estimating equations. This procedure is implemented repeatedly to obtain consistent point estimators and corresponding variance estimators simultaneously, without requiring computation of the full-data sandwich estimator. The proposed method is also well suited to modern parallel and distributed computing architectures. We illustrate its performance using real mHealth data.

In the present paper, we introduce the repeated block perturbation algorithm for large-scale longitudinal data and presents the associated theoretical results (Section 2). We then present numerical results from simulation studies (Section 3), which is followed by an analysis of real datasets (Section 4). Lastly, we provide conclusions and a discussion (Section 5). Proofs of the theoretical results are sketched in the Appendix.

## Method

### Data and Notation

Suppose we observe longitudinal or clustered data from *n* subjects or clusters. For the *i*th subject or cluster (i=1,…,n), there are $t_i$ observations with a total $n_0=\sum _{i=1}^n t_i$ observation. Let $Y_{it}$ denote the response observed at the time *t* for the subject or cluster *i*, and let $Y_{i} = (Y_{i1}, \ldots , Y_{it_i})^T$ denote the response vector for the *i*th subject or cluster. For longitudinal or clustered data, responses are assumed to be independent across subjects or clusters but correlated within each subject or cluster.

Let $X_{it}$ denote a p×1 vector of covariates corresponding to the *i*th subject or cluster at the *t*th observation, $t=1, \ldots , t_i, i=1, \ldots , n$. The relationship between response and covariates is modelled as

1 $$\begin{aligned} g (\mu _{it}) = X_{it}^T\boldsymbol{\beta } \end{aligned}$$

where $\mu _{it} = E (Y_{it}\vert X_{it})$ is the conditional mean, g(·) is a link function and $\boldsymbol{\beta }$ is a p×1 vector parameters of interest with true value $\boldsymbol{\beta }_0$. The conditional variance is specified as

$$\begin{aligned} \text {var} (Y_{it}\vert X_{it}) = \phi \nu (\mu _{it}), \end{aligned}$$

where $\nu (\mu _{it})$ is the known variance function of $\mu _{it}$ and ϕ is the scale parameter which needs to be estimated. The variance-covariance matrix of $Y_{i} = (Y_{i1}, \ldots , Y_{it_i})^T$ can be denoted by

2 $$\begin{aligned} V_i = \phi A_i^{1/2}R_i (\alpha )A_i^{1/2} \end{aligned}$$

where $A_i = \text {Diag}\left( \nu (\mu _{i1}), \ldots , \nu (\mu _{it_i}) \right)$ and $R_i (\alpha )$ represents the working correlation structure with parameter denoted by α and dimension to be $t_i\times t_i$. Note that $V_i$ will be equal to $\text {cov}(Y_i)$ if R(α) is indeed the true correlation matrix of $Y_i$. Without loss of generality, we assume $t_i = t_0$ with $t_0$ being a bounded number throughout this paper.

The generalized estimating equation (GEE) method [5] is to solve a U-function

3 $$\begin{aligned} U (\hat{\boldsymbol{\beta }}_n) = \sum _{i=1}^n D_i V_i^{-1} (Y_i - \mu _i) = 0 \end{aligned}$$

where $D_i = \frac{\partial \mu _i (\boldsymbol{\beta })}{\partial \boldsymbol{\beta }}$, and $\mu _i = (\mu _{i1}, \ldots , \mu _{it_0})^T$. With full data, ϕ can be replaced by a $n^{1/2}$-consistent estimator $\hat{\phi }_n (\boldsymbol{\beta })$ and α by a $n^{1/2}$-consistent estimator $\hat{\alpha }_n (\boldsymbol{\beta }, \phi )$. Then GEE estimator $\hat{\boldsymbol{\beta }}_n$ is essentially the solution to the equation

4 $$\begin{aligned} \sum _{i=1}^n U_i \left( \boldsymbol{\beta }, \hat{\alpha }_n (\boldsymbol{\beta }, \hat{\phi }_n (\boldsymbol{\beta })) \right) = 0. \end{aligned}$$

Under regularity conditions, one can show that $n^{1/2} (\hat{\boldsymbol{\beta }}_n-\boldsymbol{\beta }_0)$ is asymptotically normal with mean zero, and the variance-covariance matrix can be estimated by a sandwich estimator, i.e.,

5 $$\begin{aligned} n (\sum _{i=1}^n D_i^T V_i^{-1} D_i)^{-1} (\sum _{i=1}^n D_i^T V_i^{-1} \text {cov}{ (Y_i)} V_i^{-1} D_i ) (\sum _{i=1}^n D_i^T V_i^{-1} D_i)^{-1}. \end{aligned}$$

See [5] for more details. [28] also proved the almost sure existence and strong consistency of GEE estimators. Also, the sandwich variance estimator is consistent even if the correlation structure is mis-specified. If $V_i$ is indeed $\text {cov}(Y_i)$, then the variance estimator reduces to $n (\sum _{i=1}^n D_i^T V_i^{-1} D_i)^{-1}$.

### Repeated Block Perturbation Subsampling

We propose a repeated block perturbation subsampling method that reduces the sample size *n* to a smaller sample size $r_n$ via Poisson sampling, and uses these subsamples to approximate the full-data generalized estimating function. The repeated block perturbation subsampling also enables parallel computing on reduced datasets, making it suitable for the analysis of large-scale longitudinal data. The approach is summarized in the following Algorithm.

**Algorithm 1:**
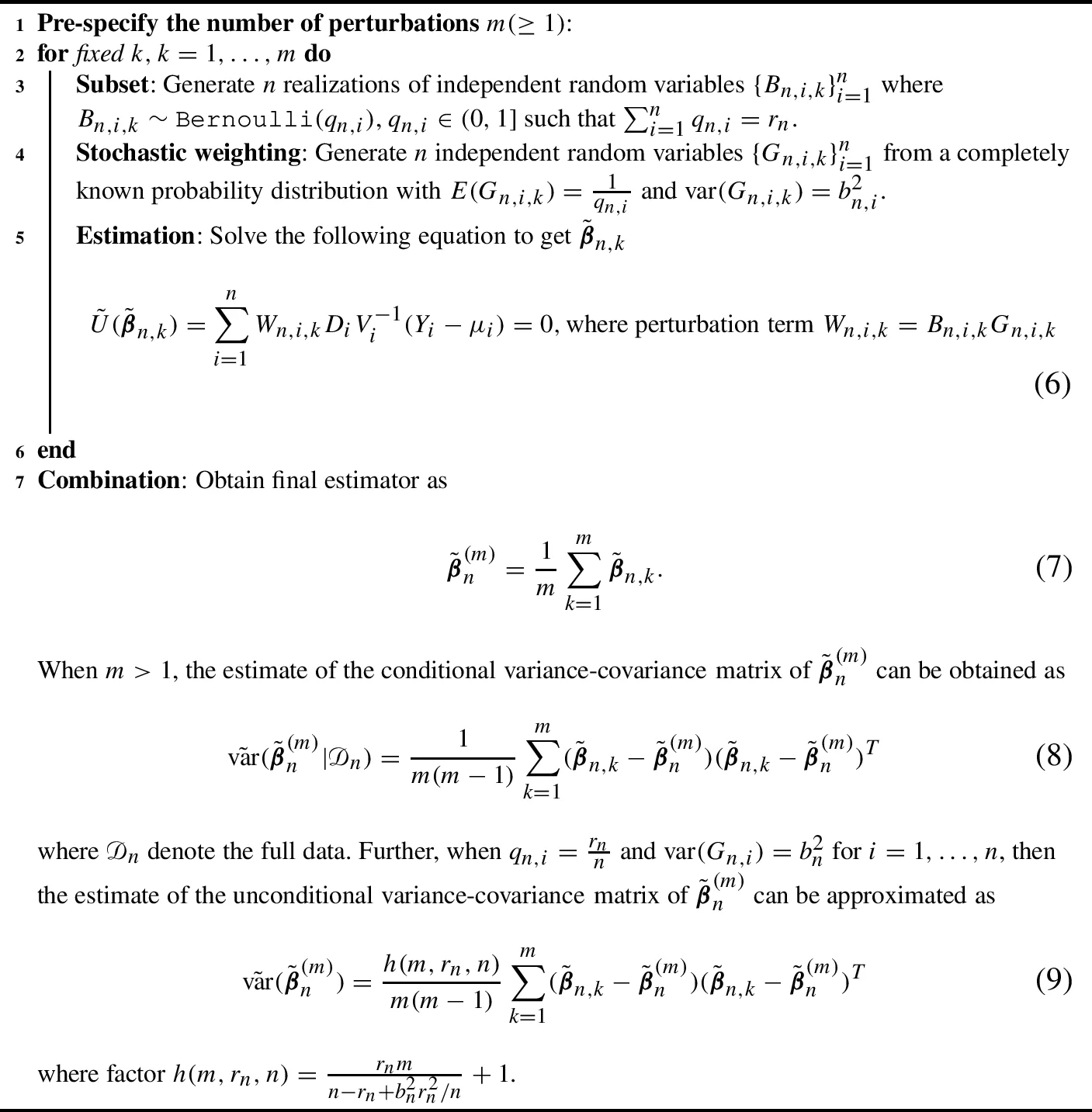
A repeated block perturbation subsampling algorithm

For each perturbation, we reduce the sample size at the subject level from *n* to $r_n$ via Poisson sampling, and then approximate the full-data estimating equation by assigning each subject-level estimating equation an independent stochastic weight.

The procedure involves the generating two different independent and identically distributed copies of a nonnegative random variable with fully specified probability distribution. Let $r_n<n$ denote the target reduced sample size at the subject level. In the first step, subsampling is performed by generating *n* independent Bernoulli random variables with inclusion probability $q_{n,i} \in (0,1]$, chosen such that $\sum _{i=1}^n q_{n,i} =r_n$. If the realization of the Bernoulli random variable equals one, all observations for the corresponding subject are included; otherwise, the subject is excluded. This subject-level sampling scheme is analogous to block bootstrap methods [29], in which resampling is also conducted at the cluster level. The resulting subsample size is random but approaching to the desired size, since the expected number of selected subjects equals $r_n$.

In the second step, we apply stochastic weighting by assigning each selected subject a nonnegative random weight with mean $\frac{1}{q_{n,i}}$ and variance $b_{n,i}^2$, for i=1,…,n. These weights are used to reweight the subject-level estimating equations so as to approximate the full-data estimating equation. Repeating this perturbation process yields a collection of subsampling-based estimators, which can be leveraged for statistical inference following the framework of Jin et al. [25] Yao and Jin [26]. Moreover, the algorithm is naturally amenable to parallel implementation under modern parallel or distributed computing architectures [11].

#### Remark 1

For the stochastic weighting in the second step, a number of probability distributions may be used to generate the random variable $G_n$. Examples include, but are not limited to, the Gamma distribution, scaled Beta distribution, and Geometric distribution. In general, if the variance of the stochastic weight $G_{n,i}$ is $b_{n,i}^2$, then the variance of the resulting weight$W_{n,i}$ is given by

$$ \text {var} (W_{n,i}) = \frac{1}{q_{n,i}}-1+b_{n,i}^2q_{n,i}, i=1,\ldots , n. $$

In particular, when $G_{n,i} \sim \texttt {Gamma} (\frac{1}{q_{n,i}},1)$, we have $\text {var} (W_{n,i}) = \frac{1}{q_{n,i}}$; when $G_{n,i} \sim \frac{2}{q_{n,i}} \texttt {Beta} (1,1)$, $\text {var} (W_{n,i}) = \frac{4}{3q_{n,i}}-1$; and when $G_{n,i} \sim \texttt {Geometric} (q_{n,i})$, $\text {var} (W_{n,i}) = \frac{2}{q_{n,i}}-2$.

A stochastic weighting variable with smaller variance introduces less randomness to the resulting estimator. In contrast, a weighting scheme with larger variance $b_n^2$ can help reduce the discrepancy between conditional variance and unconditional variance, as shown in formulas (8) and (9). The impact of different stochastic weighting choices on estimation accuracy and variance behavior is further examined in the theoretical results and simulation studies.

#### Remark 2

The procedure involves estimating nuisance parameters using reduced data for the subsample GEE estimators $\tilde{\beta }_{n,k}, k=1,\ldots , m$. Unlike full-data estimation, we instead obtain a $r_n^{1/2}$-consistent estimator $\tilde{\phi }_{r_n} (\boldsymbol{\beta })$ for ϕ and a $r_n^{1/2}$-consistent estimator $\tilde{\alpha }_{r_n} (\boldsymbol{\beta }, \phi )$ for the correlation parameter α, based on the subsample of size $r_n$. The subsample GEE estimator $\tilde{\boldsymbol{\beta }}_{n,k}$ is the solution to the equation

10 $$\begin{aligned} \sum _{i=1}^n W_{n,i,k}U_i \left( \boldsymbol{\beta }, \tilde{\alpha }_{r_n} (\boldsymbol{\beta }, \tilde{\phi }_{r_n} ( \boldsymbol{\beta })) \right) = 0. \end{aligned}$$

To solve equation (10), a modified weighted Fisher scoring algorithm can be employed, given current estimates of the correlation parameter α and the scale parameter ϕ. At each iteration, α and ϕ are updated using Pearson residuals computed from the subsample. Under the assumption that the fourth moment of the response variable is finite, these nuisance parameter estimators can be shown to be $r_n^{1/2}$ consistent due to subsampling of size $r_n$; see [5] Section 3.3 for more details. Similar to the full data estimation, substituting these $r_n^{1/2}$-consistent nuisance parameter estimators preserves the consistency and asymptotic normality of the subsample GEE estimator $\tilde{\boldsymbol{\beta }}_{n,k}$.

### Theoretical Properties

The proposed estimator can be viewed as a stochastic approximation to the full-data GEE estimator. Specifically, each subsample estimator $\tilde{\boldsymbol{\beta }}_{n,k}$ solves a weighted estimating equation in which the subject-level contributions are randomly thinned and reweighted. From this perspective, the resulting variance expressions can be interpreted as modifications of the classical sandwich variance estimator for GEE. The additional multiplicative factors arise from two sources: (i) the randomness induced by subsampling (through $B_{n,i,k}$), and (ii) the additional variability introduced by stochastic weighting (through $G_{n,i,k}$). When the subsample size $r_n$ is close to *n* and the variability of the weights is small, these expressions reduce to the standard sandwich form. When $r_n \ll n$, the additional scaling factors quantify the efficiency loss due to subsampling and provide a principled way to recover valid variance estimates.

The following result summarizes both consistency and asymptotic normality of the proposed estimator. For clarity, we state the main result in a compact form and defer technical details to the Appendix.

#### Theorem 1

(Consistency and asymptotic normality)

(i) Under the mild regularity assumptions, the global maximum of $\tilde{l} (\boldsymbol{\beta },y,W_n)$ is a consistent estimate of full data GEE estimator $\hat{\boldsymbol{\beta }}_n$ condition on the data, and the global maximum of $\tilde{l} (\boldsymbol{\beta },Y,W_n)$ is a consistent estimate of $\boldsymbol{\beta }_0$ as $r_n, n \rightarrow \infty$.

(ii) Condition on data, under assumptions on higher-order moment conditions, $(r_nm)^{1/2} (\tilde{\boldsymbol{\beta }}^{ (m)}_n - \hat{\boldsymbol{\beta }}_n)$ is asymptotically multivariate Gaussian with zero mean and variance-covariance matrix given by

11 $$\begin{aligned} \begin{aligned} \lim _{n \rightarrow \infty } r_n&\left( \sum _{i=1}^n (Q_i (\hat{\boldsymbol{\beta }}_n) + D_i (\hat{\boldsymbol{\beta }}_n)^TV_i (\hat{\boldsymbol{\beta }}_n)^{-1}D_i (\hat{\boldsymbol{\beta }}_n) )\right) ^{-1}\\ \times&\left( \sum _{i=1}^n (\frac{1}{q_{n,i}}-1+b_{n,i}^2q_{n,i}) D_i (\hat{\boldsymbol{\beta }}_n)^T V_i (\hat{\boldsymbol{\beta }}_n)^{-1} (Y_i - \mu _i(\hat{\boldsymbol{\beta }}_n) )^2V_i (\hat{\boldsymbol{\beta }}_n)^{-1}D_i (\hat{\boldsymbol{\beta }}_n) \right) \\ \times&\left( \sum _{i=1}^n (Q_i (\hat{\boldsymbol{\beta }}_n) + D_i (\hat{\boldsymbol{\beta }}_n)^TV_i (\hat{\boldsymbol{\beta }}_n)^{-1}D_i (\hat{\boldsymbol{\beta }}_n)) \right) ^{-1} \end{aligned} \end{aligned}$$

as $r_n, n \rightarrow \infty$, and $Q_i$ is a p×p matrix given by

$$\begin{aligned} Q_i (\boldsymbol{\beta }) = \frac{\partial D_i (\boldsymbol{\beta }) V_i^{-1} (\boldsymbol{\beta })}{\partial \boldsymbol{\beta }^T} (Y_i-\mu _i({\boldsymbol{\beta }})). \end{aligned}$$

The variance expression in (11) can be viewed as a modified sandwich estimator. The outer matrices correspond to the inverse of the sensitivity matrix, while the middle term captures variability of the estimating equations. Compared to the classical GEE sandwich variance in (5), the additional factor $(\frac{1}{q_{n,i}} - 1 + b_{n,i}^2 q_{n,i})$ accounts for the extra variability induced by subsampling and stochastic weighting. When $q_{n,i} \approx 1$ and $b_{n,i}^2$ is small, this term approaches 1 and the expression reduces to the standard sandwich form. Although the analytical expression appears complex, its computation only involves subject-level quantities already available in standard GEE implementations, and can be efficiently computed in parallel across perturbations.

#### Remark 3

In practice, the choice of subsample size $r_n$, number of perturbations *m*, and weighting distribution affects both computational cost and statistical efficiency. A larger $r_n$ reduces approximation error but increases computational burden, while a smaller $r_n$ improves scalability at the cost of higher variance. The number of perturbations *m* controls Monte Carlo variability; moderate values (e.g., m=50–200) are typically sufficient in our experiments. For stochastic weighting, distributions with smaller variance (e.g., Gamma) tend to yield more stable estimates, whereas larger variance can improve agreement between conditional and unconditional variance estimators. In practice, we recommend starting with Gamma weights and adjusting based on computational and inferential goals. Overall, we recommend using the empirical variance estimator in (8) together with the scaling factor in (9) for routine implementation.

The proposed method is developed under the standard GEE asymptotic framework where the number of clusters *n* diverges and cluster size remains bounded. Therefore, the theoretical guarantees may not directly extend to settings with a small number of clusters and large within-cluster observations. The method inherits the robustness properties of GEE with respect to correlation misspecification. However, like standard GEE estimators, it may be sensitive to extreme outliers. Incorporating robust estimating equations within the proposed subsampling framework is a promising direction for future work.

## Simulation Study

This section presents simulation studies on the finite sample performance of the proposed estimator and its associated variance estimator derived from Algorithm 1. We first considered a generalized linear model with the identity link function,

12 $$\begin{aligned} \mu _{it} = \boldsymbol{X}_{it}^T \boldsymbol{\beta } = \beta _0 + X_{i1t}\beta _1 + X_{i2t}\beta _2 \end{aligned}$$

where $t=1, \ldots , 10, i =1, \ldots , 10000$, and $\boldsymbol{\beta }_0 = (1,1,1)^T$ with p=3.

For each subject *i*, the covariate vector $\boldsymbol{X}_i^T = (X_{i,1}, \ldots , X_{i,10})$, corresponding to the second and third regression coefficients, was generated from a multivariate distribution with mean $(0.1, 0.2, \ldots , 1.0)^T$ and a finite covariance. Specifically, at each time point t=1,…,10, the partial design matrix $\boldsymbol{X}_{n\times (p-1)}$, excluding the intercept, was generated from either multivariate normal $N (\boldsymbol{0.1} t, \boldsymbol{\Sigma })$ or multivariate *t*-distribution with 3 degree of freedom and the same mean and covariance structure. The covariance matrix Σ was specified as $\Sigma _{i,j} = 0.01\times 0.5^{|i-j|}$ for i,j=1,…,10. The dispersion parameter ϕ was set to 1, and three working correlation structures were considered: independence, exchangeable and AR-1 correlation matrices with α=0.3 and α=0.7.

We also examined a generalized linear model with a logit link

13 $$\begin{aligned} \text {logit} (E (Y_{it})) = \beta _0 + \beta _1 X_{1i} + \beta _2 X_{2i} \end{aligned}$$

where $X_1 \sim \texttt {Bernoulli} (0.2)$, $X_2 \sim \texttt {Bernoulli} (0.3)$, $t=1, \ldots , 3, i =1, \ldots , 10,000,$ and $\boldsymbol{\beta }_0 = (0,1,1)^T$. Independence, exchangeable and AR-1 correlation matrices with α=0.3 were considered. In addition, a rare-event scenario was investigated by setting $X_1 \sim \texttt {Bernoulli} (0.01)$.

During the perturbation subsampling step, the sampling probabilities were defined as

$$ q_{n,i} = I(X_{1,i}=1) + \frac{r_n - 100}{n-100}I(X_{1,i}=0) $$

where n=10000, ensuring that all rare-event observations were included in the subsample.

To investigate the perturbation estimator with different stochastic weights, we compared the following estimators

1. pooled estimator (full data estimator) (Full);
2. uniform subsampling estimator based on sampling with replacement (unisMUL);
3. uniform subsampling estimator based on sampling without replacement (unisGEOM);
4. perturbation estimator based on Gamma$(\frac{1}{q_n},1)$ distribution (gammaPERT);
5. perturbation estimator based on $\frac{2}{q_n}$Beta (1, 1) distribution (betaPERT);
6. perturbation estimator based on Geometric$$ (q_n)$$ distribution (geomPERT)

where $$q_n = \frac{r_n}{n}$$.

To investigate the effect of different reduced sample sizes, we computed the estimators using subsample size $r_n$ ranging from 200, 300, 500, 800, 1000, 1200, 1500, 2000, 3000 to 5000. To assess the impact of the number of perturbations, the number of repetitions *m* was set to 20, 50, 200, 500.

Across N=1000 simulation replicates, we evaluated estimation accuracy using both conditional and unconditional empirical mean squared errors (MSE), defined as $MSE_c = \frac{1}{N}\sum _{i=1}^N ||\tilde{\boldsymbol{\beta }}_{n,i}-\hat{\boldsymbol{\beta }}_n||^2\text { and } MSE_{unc} = \frac{1}{N}\sum _{i=1}^N ||\tilde{\boldsymbol{\beta }}_{n,i}-\boldsymbol{\beta }_0||^2$ where $\tilde{\boldsymbol{\beta }}_{n,i}$ denotes the estimator from the *i*th simulation replicate. Empirical coverage probabilities based on both conditional and unconditional confidence intervals were used to assess the performance of the proposed variance estimators.

For each simulated dataset, the subsample perturbation estimators were computed using the geepack package from R [30], with weights argument specified as the perturbation weights and the std.err argument set to “san.se”. ll computations were performed on a high-performance computing (HPC) cluster running Linux (CentOS 7.6.1810) in the Department of Systems Biology at Columbia University.

**Fig. 1:**
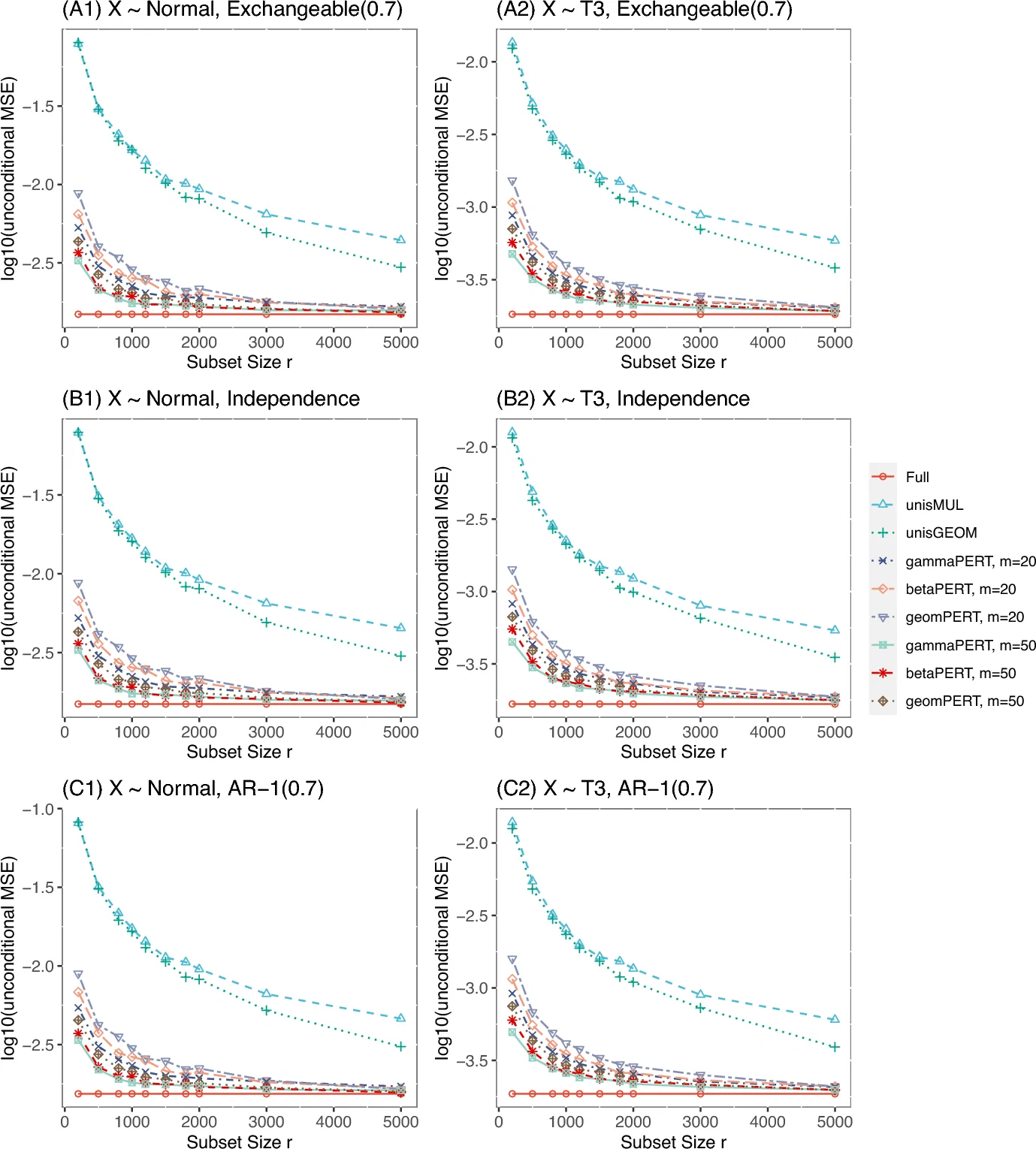
Comparison of empirical unconditional MSEs between repeated perturbation sampling with m=20,50 and other subsampling methods for generalized linear regression model with identity link with α=0.3 based on N=1000 simulations

**Fig. 2:**
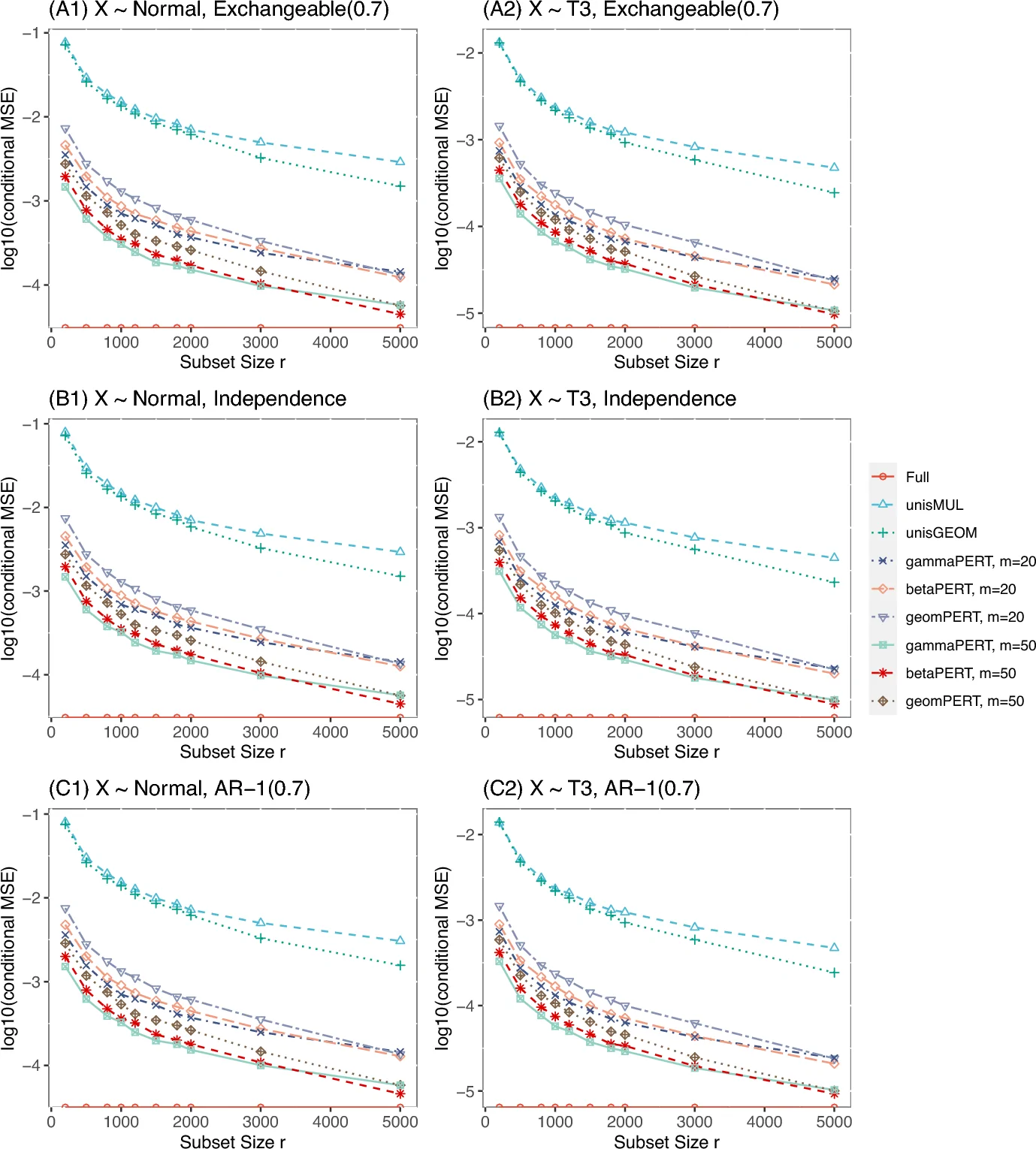
Comparison of empirical conditional MSEs between repeated perturbation sampling with m=20,50 and other subsampling methods for generalized linear regression model with identity link with α=0.3 based on N=1000 simulations

**Fig. 3:**
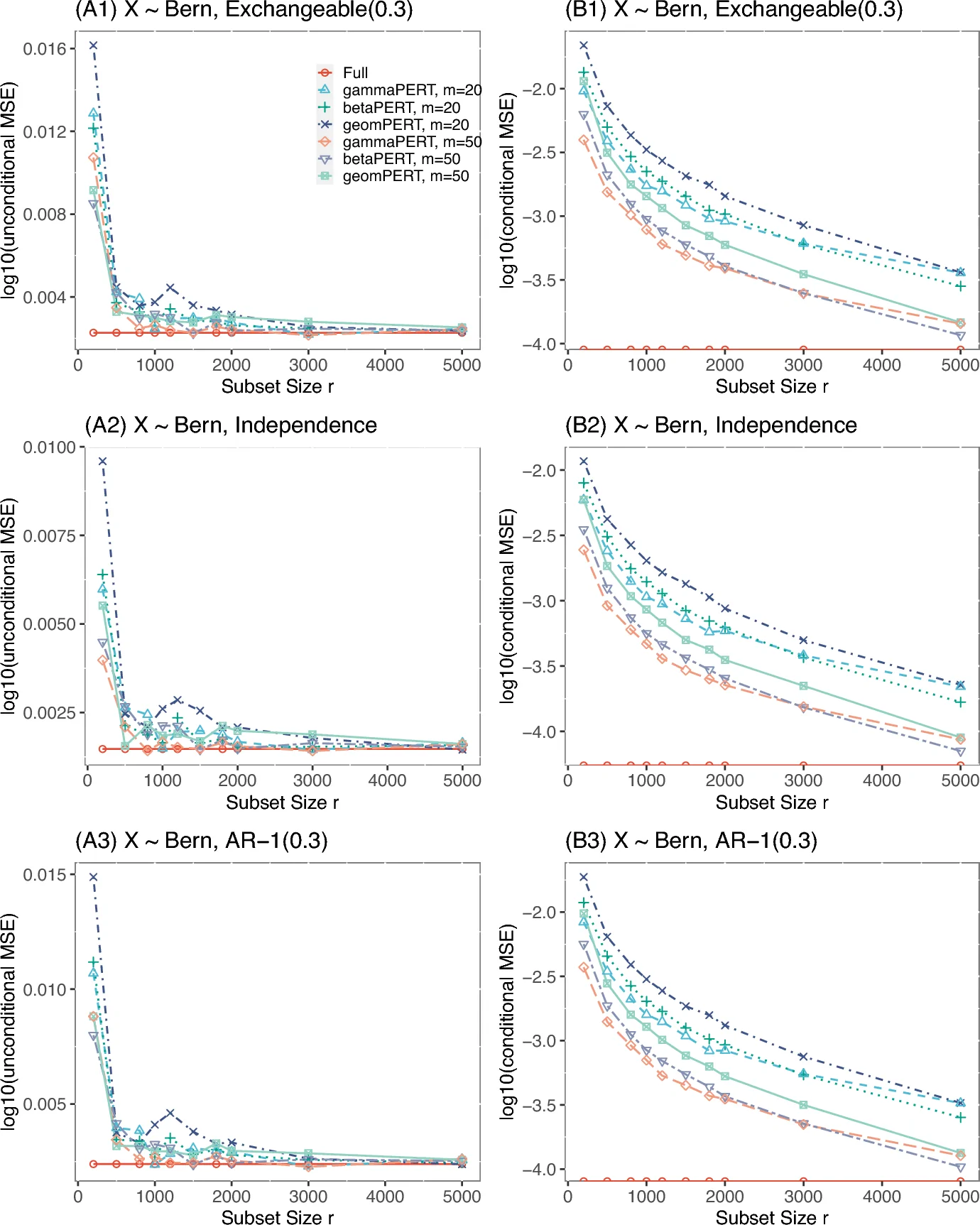
Comparison of empirical MSEs between repeated block perturbation subsampling with m=20,50 and other subsampling methods for generalized linear regression model with logit link with α=0.3 based on N=1000 simulations

**Fig. 4:**
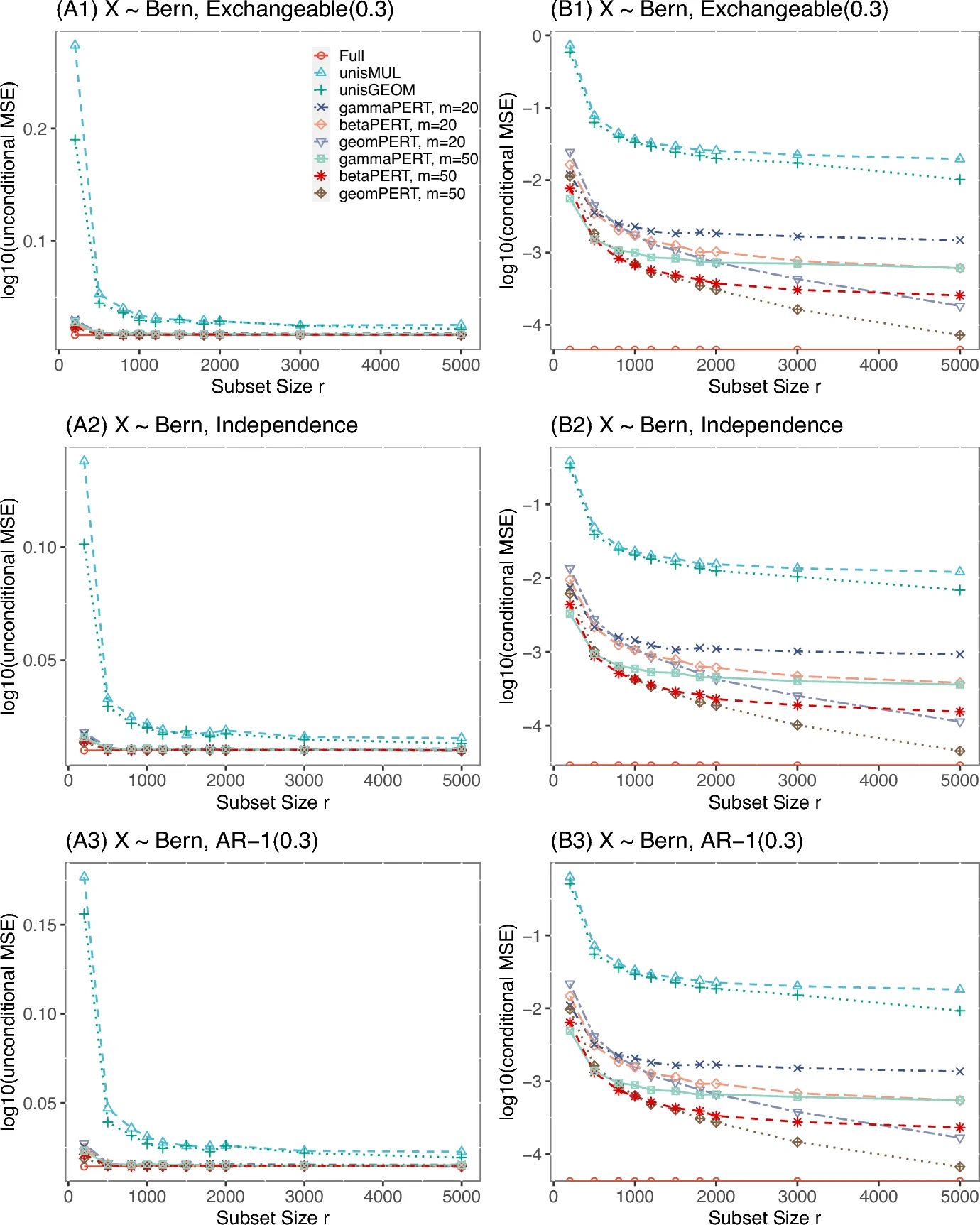
Comparison of empirical MSEs between repeated block perturbation subsampling with m=20,50 and other subsampling methods for generalized linear regression model with logit link with α=0.3 for rare events based on N=1000 simulations

**Table 1:** Computing time of repeated perturbation methods with m=50 compared to other subsampling methods. The CPU time is the average of M=1000 simulations across all subsample sizes and four scenarios

Method	unisMUL	unisGEOM	gammaPERT	betaPERT	geomPERT
CPU time(s)	0.027	0.026	0.009	0.010	0.014

**Fig. 5:**
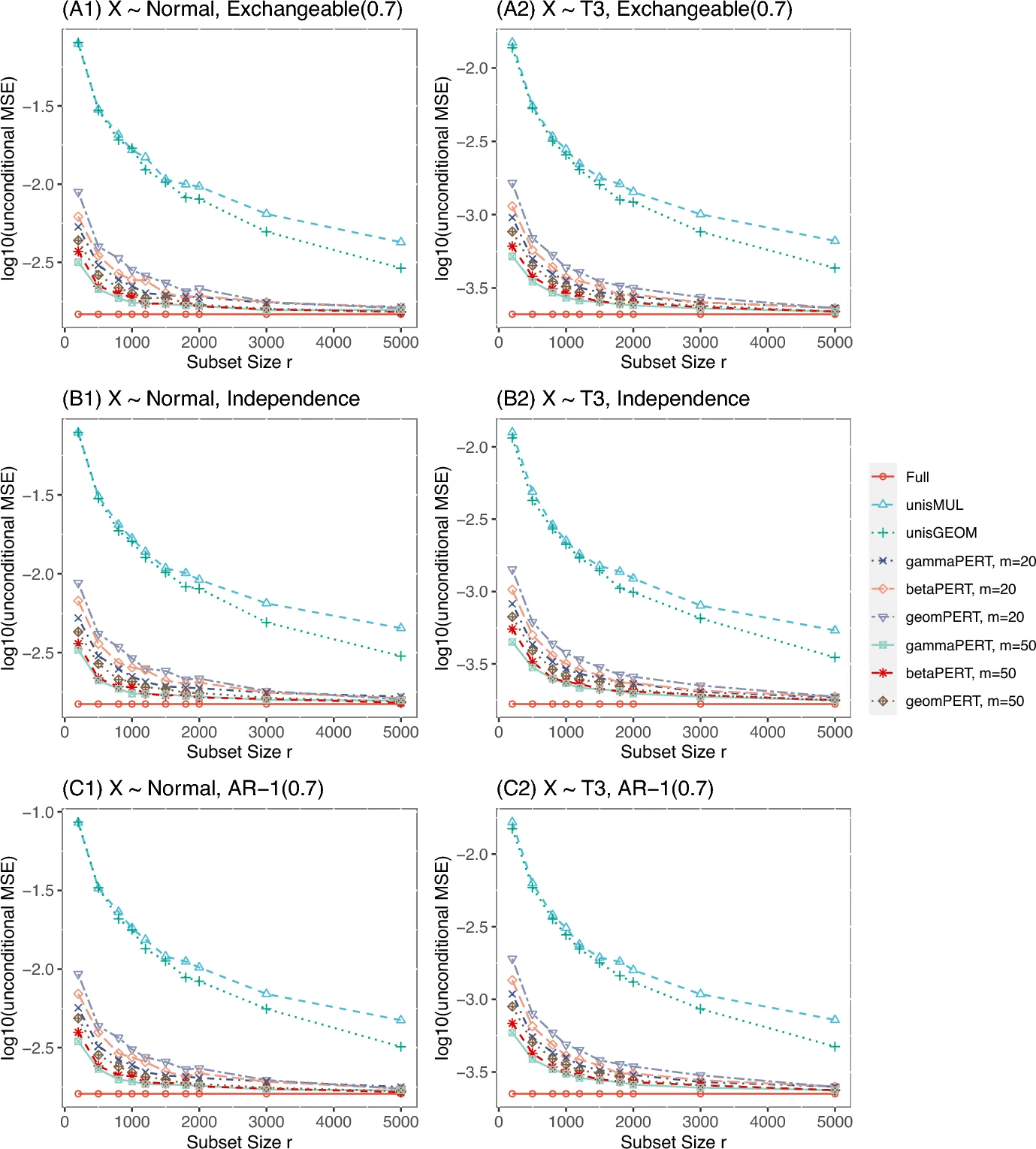
Comparison of empirical unconditional MSEs between repeated block perturbation subsampling with m=20,50 and other subsampling methods for generalized linear regression model with identity link with α=0.7 based on M=1000 simulations

**Fig. 6:**
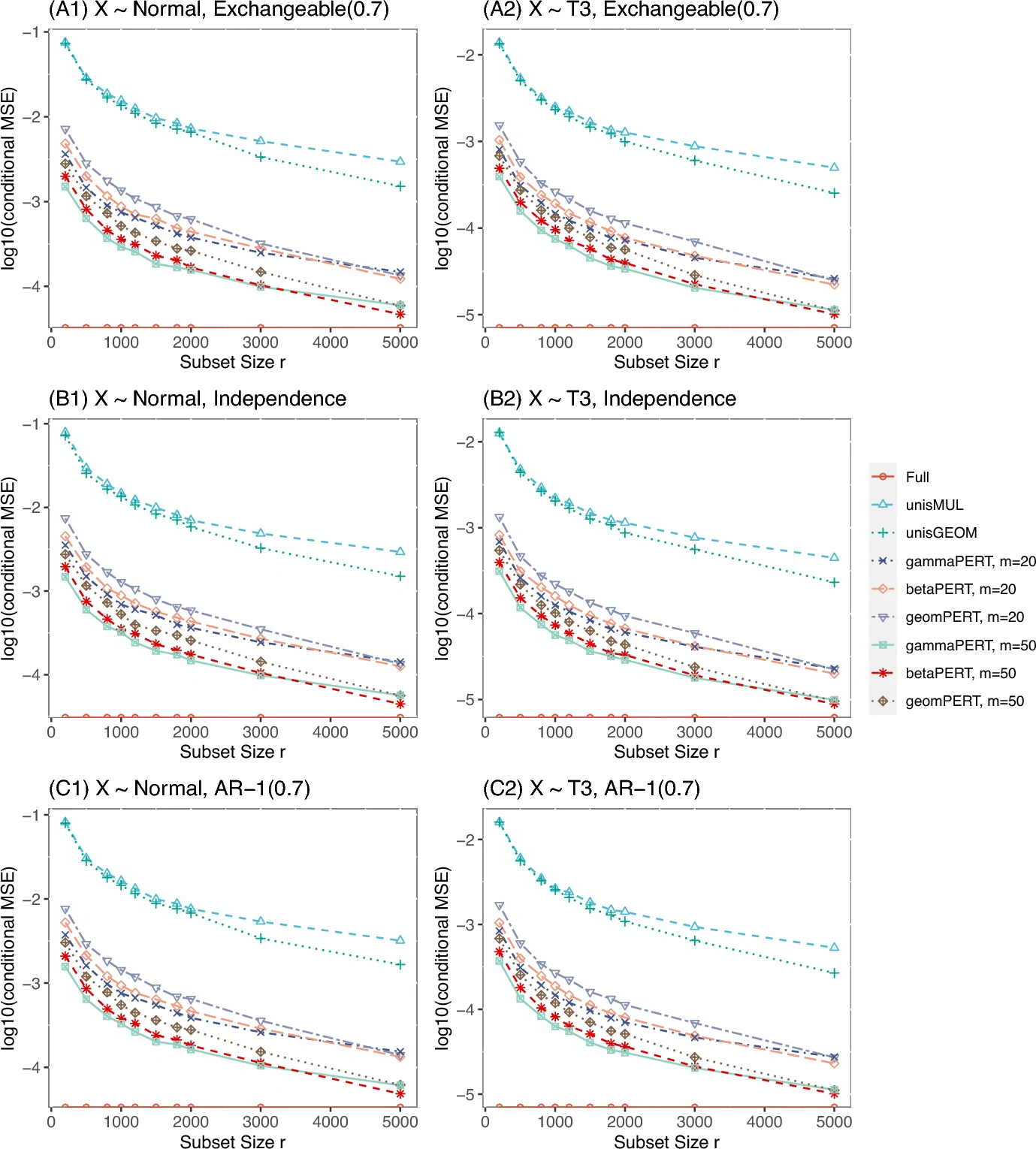
Comparison of empirical conditional MSEs between repeated block perturbation subsampling with m=20,50 and other subsampling methods for generalized linear regression model with identity link with α=0.7 based on N=1000 simulations

The Figure 1 and Figure 2 present the performance of the repeated block perturbation estimator compared with existing methods for the generalized linear regression model with the identity link and correlation parameter α=0.3. For all subsampling methods and under different design matrices, both the conditional and unconditional MSEs decreased as the subsample size increased. The perturbation estimators achieved substantially smaller MSEs by averaging over m=20 perturbations, and increasing *m* from 20 to 50 further reduced the approximation error. In addition, the repeated block perturbation subsampling methods required less computational time than the uniform subsampling methods based on sampling with or without replacement (Table 1). Similar performance patterns were observed when the correlation parameter increased to α=0.7 (Figure 5 and Figure 6).

Similar trends were also observed for the generalized linear regression model with the logit link. As shown in Figure 3, the efficiency of the repeated block perturbation estimator was very close to that of the full-data estimator in terms of unconditional MSE when the subsample size exceeded 1000. Results for the logistic regression model with rare events are shown in Figure 4. In this setting, the block perturbation estimator remained close to the full-data estimator in terms of unconditional MSE, while the conditional MSE exhibited a slower rate of decrease once the subsample size exceeded 2000. This behavior reflects the fact that including all rare events in the subsample already provides sufficient information at relatively small subsample sizes. Moreover, among the perturbation estimators, geomPERT–which has the largest $b_n^2$—achieved the smallest conditional MSE compared with gammaPERT and betaPERT for the same number of perturbations. This indicates that a stochastic weighting scheme with larger variance can help mitigate bias in rare-event scenarios.

Finally, we evaluated the performance of the proposed variance estimators for the three perturbation methods using m=50,200,500 based on formulas  (8) and formula (9). Table 5 and Table 6 report the coverage probabilities for the generalized linear regression model with identity link and α=0.3. When the design matrix followed a normal distribution, the empirical coverage of the full-data estimator based on the proposed conditional variance estimator in Algorithm 1 was closer to the nominal 0.95 level than when the design matrix followed a *t*-distribution. Furthermore, increasing the number of perturbations did not always improve conditional variance performance under the *t*-distributed design, a phenomenon consistent with existing results in the divide-and-conquer literature, which suggest that the number of partitions should be smaller than the sample size within each partition [11, 31–36]. To ensure that the conditional bias remains negligible relative to the conditional variance for valid inference, we recommend using $m \le r_n/10$ in practice. We further evaluated the unconditional variance estimator (Table 6). The empirical coverage probabilities for the true parameter values based on the proposed unconditional variance estimator in Algorithm 1 were close to the nominal 0.95 level for all three perturbation methods. Similar patterns were observed for the generalized linear regression model with the logit link, with results summarized in Table 7 and Table 8.

## Application

### SHMAS Dataset

In this section, we applied our proposed procedure to data from the SleepHealth Mobile App Study (SHMAS) data [37]. The objective of SHMAS was to investigate the relationship between sleep habits and daytime functioning. The study was launched on the Apple App Store on March 2, 2016, and a total of 12,356 participants provided informed consent to participate in the study through June 26, 2019. Participants who agreed to share data broadly were followed for an average of 935.4 days (SD=355.1, range=1-1212 days) [37]. Due to the substantial heterogeneity in app usage across participants, we restricted the analysis to the first three sleep records of each subject. Only complete cases were included. This resulted in a total of 1,311 subjects (3,933 repeated measures) for the sleep quality dataset and 1,408 subjects (4,224 repeated measures) for the sleep check dataset.

To examine the association between sleep habits and daytime functioning, the following covariates were incorporated into the model:

1. Gender (1=female, 2=male);
2. Income (USD) (1=less than 10000, 2=10000-49999, 3=50000-99999, 4=100,00-149999, 5=150000-199999, 6=200000-249999, 7=250000 and above);
3. Marital (1=married, 0=not married);
4. Work (1=employed, 0=not employed);
5. Good life (1=strongly disagree, 2=disagree, 3=neither agree nor disagree, 4=agree; 5=strongly agree);
6. Smoke (1=smoking, 0=not smoking);
7. Caffeine (number of caffeinated beverages (cups/cans) each day);
8. Alcohol (alcoholic beverages such as beer (12 oz.), wine (5 oz.) in a typical week).

**Fig. 7:**
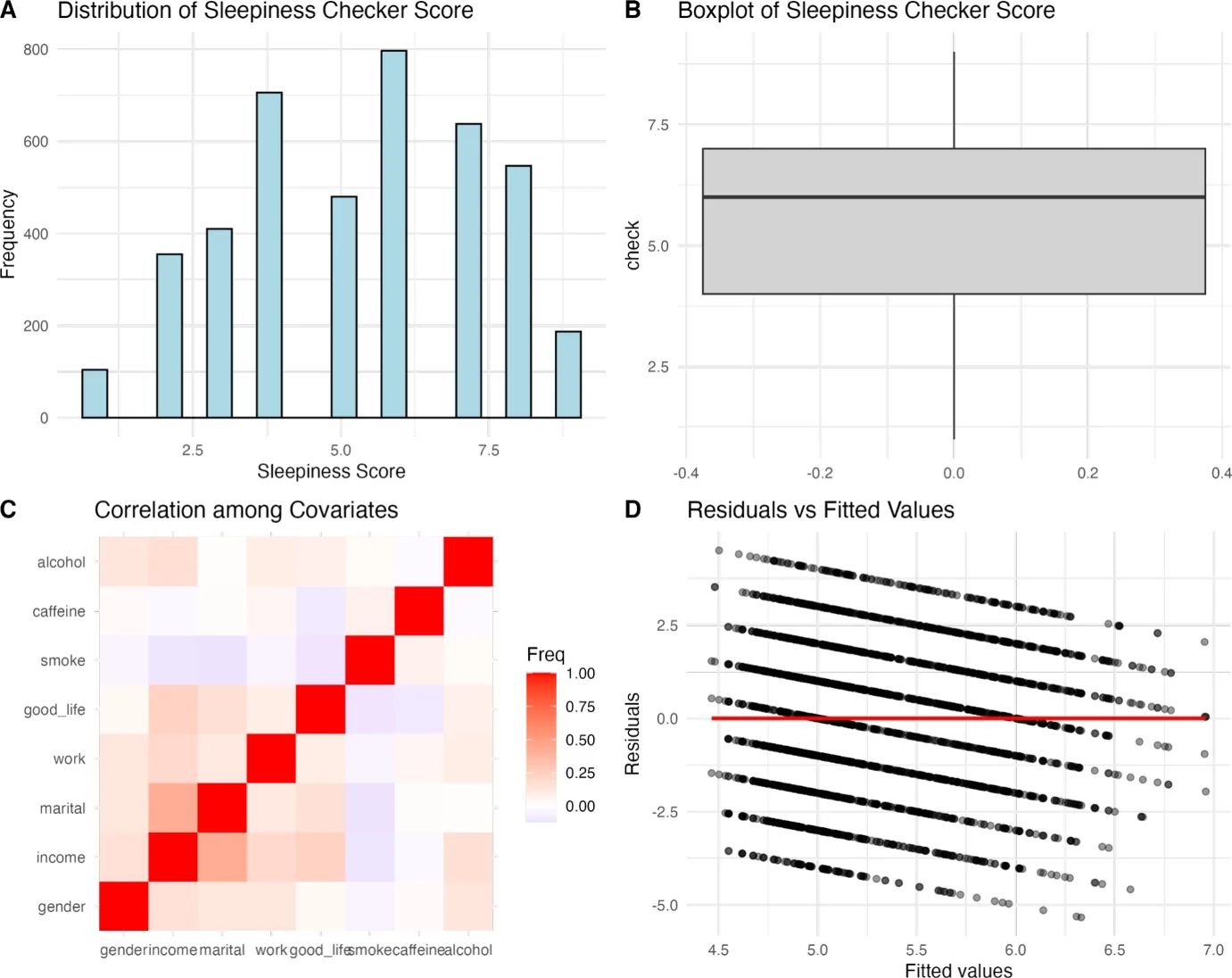
Descriptive analysis of the sleepiness check score in SHMAS dataset

We first examine the marginal distribution of the response variables. Figure 7-A shows the histogram of the sleepiness checker score. The distribution appears approximately continuous with moderate skewness and no excessive concentration at boundary values, suggesting that treating the outcome as continuous is reasonable. We also examined potential outliers using boxplot (Figure 7-B) and standardized residuals (Figure 7-D). In addition, we explored pairwise relationships among explanatory variables (Figure 7-C). The correlations between covariates were low to moderate (min: 0.02, max: 0.42), and no evident time effect were observed across the three measurement time points. No strong nonlinear interactions were observed, and therefore we adopt a main-effects model in the analysis. More flexible specifications could be considered in future work. Based on this empirical distribution, we adopt a Gaussian working model with an identity link function, which allows direct interpretation of regression coefficients.

We examined the effect of demographic factors on the sleepiness checker score (1=extremely alert, 2=very alert, 3=rather alert, 4=alert, 5=neither alert nor sleepy, 6=some signs of sleepiness, 7=sleepy, but no difficulty remaining awake, 8=sleepy,some effort to keep alert, 9=extremely sleepy, fighting sleep) using the following generalized linear regression model with identity link, i.e., for *i*th subject with *t*th repeated measure,

14 $$\begin{aligned} \begin{aligned} \text {sleepiness checker score}_{it}&= \beta _0 \!+\! \beta _1 \text {Gender}_{i} \!+\! \beta _2 \text {Income}_{i} + \beta _3 \text {Marital}_{i} + \beta _4 \text {Work}_{i} \\&\quad + \beta _5 \text {Good Life}_{i} + \beta _6 \text {Smoke}_{i} + \beta _7 \text {Caffeine}_{i}\\  &\quad + \beta _8 \text {Alcohol}_{i} + \text {error}_{it}. \end{aligned} \end{aligned}$$

**Table 2:** Estimation of coefficient and standard error for for SHMAS dataset using generalized linear regression model with natural link for sleepiness check. Results from the full dataset and repeated perturbation algorithms with m=20 and $r_n=500$ are presented

Variables	Full		gammaPERT			betaPERT			geomPERT		
	Coef.	SE	Coef.	Cond. SE	SE	Coef.	Cond. SE	SE	Coef.	Cond. SE	SE
Intercept	7.745	0.244	7.734	0.101	0.298	7.812	0.103	0.308	7.728	0.153	0.409
Gender	-0.788	0.084	-0.775	0.039	0.113	-0.825	0.034	0.102	-0.809	0.043	0.115
Income	-0.053	0.031	-0.039	0.013	0.039	-0.033	0.013	0.038	-0.021	0.017	0.044
Marital	-0.082	0.091	-0.106	0.038	0.112	-0.036	0.040	0.119	-0.068	0.039	0.105
Work	-0.067	0.098	-0.052	0.026	0.076	-0.084	0.043	0.128	-0.054	0.053	0.141
Good life	-0.191	0.051	-0.197	0.023	0.066	-0.220	0.019	0.057	-0.207	0.032	0.086
Smoke	0.249	0.129	0.203	0.058	0.171	0.264	0.058	0.173	0.307	0.086	0.230
Caffeine	-0.001	0.008	-0.007	0.003	0.007	0.007	0.005	0.014	0.001	0.006	0.016
Alcohol	-0.070	0.054	-0.042	0.018	0.054	-0.050	0.023	0.069	-0.117	0.033	0.089

**Table 3:** Estimation of regression coefficient for SHMAS dataset using generalized linear regression model with logit link for sleep quality. Results from the full dataset and repeated perturbation algorithms with m=20 and $r_n=500$ are presented

Variables	Full		gammaPERT			betaPERT			geomPERT		
	Coef.	SE	Coef.	Cond. SE	SE	Coef.	Cond. SE	SE	Coef.	Cond. SE	SE
Intercept	-0.250	0.287	-0.056	0.122	0.357	-0.185	0.073	0.219	-0.267	0.123	0.330
Gender	0.355	0.104	0.327	0.033	0.097	0.339	0.037	0.112	0.323	0.047	0.126
Income	0.009	0.035	0.015	0.011	0.033	0.013	0.011	0.034	0.026	0.022	0.059
Marital	0.025	0.113	0.012	0.043	0.126	0.069	0.041	0.124	-0.008	0.043	0.115
Work	0.213	0.119	0.161	0.033	0.095	0.155	0.035	0.106	0.153	0.050	0.133
Good life	0.251	0.055	0.228	0.028	0.083	0.245	0.023	0.068	0.261	0.031	0.083
Smoke	-0.264	0.163	-0.238	0.069	0.201	-0.259	0.062	0.185	-0.115	0.076	0.203
Caffeine	0.010	0.009	0.006	0.003	0.009	0.014	0.004	0.012	0.016	0.005	0.013
Alcohol	-0.080	0.068	-0.065	0.021	0.061	-0.071	0.029	0.086	-0.063	0.028	0.075

Table 2 summarizes the analysis results. Using the full dataset, male sex and good life were significantly associated with alertness and no sleepiness (gender: -0.800, SE 0.084, p-value < 0.001; good life: -0.190, SE 0.051, p-value < 0.001). The proposed repeated block perturbation subsampling with $r_n = 500$ and m=20 produced coefficient estimates that were highly consistent with those obtained from the full data analysis. The conditional standard errors from the perturbation estimators were approximately $\sqrt{g(r_n,n)/ (r_n m)}$ times the full data standard errors (0.38 for gammaPERT, 0.37 for betaPERT, 0.43 for geomPERT), in agreement with the theoretical results in Theorem 1. These findings indicate that the point estimates were stable across different subsample perturbations. In addition, the unconditional variances of the perturbation estimators were approximately $t(m,r_n,n)/n$ times the variances of full data estimates (1.14 for gammaPERT, 1.14 for betaPERT, 1.18 for geomPERT), consistent with the factor in Theorem A.3.

To illustrate the use of the proposed approach in generalized linear regression model with logit link, we analyzed the effect of demographic factors on the sleep quality checker score, i.e.,

15 $$\begin{aligned} \begin{aligned} \text {logit} (Pr\{Y_{it}=1\})&= \beta _0 + \beta _1 \text {Gender}_{i} + \beta _2 \text {Income}_{i} + \beta _3 \text {Marital}_{i} + \beta _4 \text {Work}_{i} + \\&\beta _5 \text {Good Life}_{i} + \beta _6 \text {Smoke}_{i} + \beta _7 \text {Caffeine}_{i} + \beta _8 \text {Alcohol}_{i} \end{aligned} \end{aligned}$$

where Y=1 representing sleep quality was fair to very good, and Y=0 indicating poor sleep quality. Table 3 presents the analysis results. The full-data analysis showed that male sex and good life again were significantly associated with good sleep quality (gender: 0.355, SE 0.104, p-value < 0.001; good life: 0.251, SE 0.055, p-value < 0.001). The proposed repeated block perturbation subsampling with $r_n = 500$ and m=20 yielded results that were highly consistent with those obtained from the full dataset. The conditional standard errors were stable across different subset perturbations, and were approximately $\sqrt{g(r_n,n)/ (r_n m)}$ times the standard errors with the full dataset (0.362 for gammaPERT, 0.353 for betaPERT, 0.403 for geomPERT). The unconditional variances of perturbation estimates were approximately $t(m,r_n,n)/n$ times the variances of the estimates obtained with the full dataset (1.13 for gammaPERT, 1.12 for betaPERT, 1.16 for geomPERT). Notably, the computational complexity was reduced from $O (n p^2)$ to $O (r_n p^2)$, with substantially lower memory requirements compared to the full-data analysis.

The conditional MSE and conditional coverage rates were evaluated using N=1000 different subsample drawn from the full dataset across a range of subsample sizes (Table 9). The repeated block perturbation subsampling method consistently achieved smaller conditional MSEs than the uniform subsampling approaches. In addition, the proposed conditional variance estimators demonstrated satisfactory coverage of the full-data estimates (Table 10).

### UK Biobank Dataset

We further applied the proposed procedure to data from the UK Biobank [38], a large prospective cohort study that has followed the health of approximately 500, 000 volunteer participants aged between 40-69 years at recruitment between 2006 and 2010 (https://www.ukbiobank.ac.uk). Physical activity was measured using a wrist-worn accelerometer.The primary objective of this analysis was to examine the association between weekday accelerometer wear time and demographic characteristics. A total of 82, 670 participants with complete data across five repeated measurements were included in the analysis. The following covariates were incorporated into the model:

1. Age;
2. Gender (1=female, 0=male);
3. Education (1=college or university degree, 0=others);
4. Health status (1=good to excellent, 0=poor to fair);
5. Smoke (1=previous or current, 0=never);
6. Alcohol (1=previous or current, 0=never).

In the UK Biobank analysis, subjects with missing observations were excluded in the given dataset. The resulting response variable can be interpreted as the observed duration of participation among individuals with complete data. This approach implicitly assumes that missingness is independent of the outcome conditional on observed covariates. We acknowledge that this is a simplifying assumption and that more sophisticated approaches could be incorporated within our framework. Extensions of the proposed method to incorporate inverse probability weighting or multiple imputation would be of interest for future work with adherence of the study considered.

We examined the effects of these demographic factors on weekday accelerometer wear time using a generalized linear regression model with identity link. Specifically, for the *i*th subject at the *t*th repeated measure time, the model was specified as

16 $$\begin{aligned} \begin{aligned} \text {weekday accelerometer wear time}_{it} =&\beta _0 + \beta _1 \text {age}_{i} + \beta _2 \text {gender}_{i} + \beta _3 \text {education}_{i} +\\ \beta _4 \text {health status}_{i} +&\beta _5 \text {smoke}_{i} + \beta _6 \text {alcohol}_{i} + \text {error}_{it}. \end{aligned} \end{aligned}$$

**Table 4:** Estimation of coefficient and standard error for for UK biobank dataset using generalized linear regression model with natural link for acceleration wear time. Results from the full dataset and repeated perturbation algorithms with m=50 and $r_n=5,000$ are presented

Variables	Full		gammaPERT			betaPERT			geomPERT		
	Coef.	SE	Coef.	Cond. SE	SE	Coef.	Cond. SE	SE	Coef.	Cond. SE	SE
Intercept	19.065	0.092	19.058	0.055	0.111	18.999	0.065	0.119	19.122	0.061	0.099
Age	0.044	0.001	0.045	0.001	0.001	0.045	0.001	0.002	0.044	0.001	0.001
Gender	-0.082	0.019	-0.079	0.012	0.023	-0.085	0.011	0.020	-0.067	0.014	0.023
Education	-0.003	0.019	0.000	0.011	0.021	-0.022	0.012	0.022	-0.009	0.012	0.020
Health	0.277	0.026	0.272	0.018	0.037	0.279	0.016	0.030	0.289	0.021	0.034
Alcohol	0.000	0.056	-0.019	0.030	0.060	0.031	0.042	0.077	-0.036	0.043	0.070
Smoke	-0.146	0.019	-0.153	0.011	0.022	-0.142	0.011	0.020	-0.135	0.015	0.024

Table 4 reports the analysis results. The full-data analysis indicated that older age, male sex, better health status, and non-smoking status were significantly associated with longer weekday accelerometer wear time (age: 0.044, SE 0.001, p-value < 0.001; gender: -0.082, SE 0.019, p-value < 0.001; health status: 0.277, SE 0.026, p-value < 0.001; smoke status: -0.146, SE 0.019, p-value < 0.001). The proposed repeated block perturbation subsampling approach with $r_n = 5000$ and m=50 produced coefficient estimates and standard errors that closely matched those obtained from the full-data analysis.

In terms of computational efficiency, the full-data analysis required approximately 11 seconds of CPU time, whereas the repeated block perturbation algorithm required on average 1 second with parallel computing. In addition, the perturbation-based approach exhibited substantially lower memory requirements than the full-data analysis.

## Discussion

In this paper, we proposed a novel repeated block perturbation subsampling method based on two classes of stochastic weighting variables for the analysis of large-scale longitudinal data. Finite sample simulation studies and real data applications demonstrate that the proposed approach can simultaneously produce valid point estimators and variance estimators with substantially reduced computational cost. Although stochastic weighting variables with smaller variance tend to yield estimators with lower variance, our results show that weighting schemes with larger variance can effectively mitigate bias, particularly in rare-event settings, as illustrated in the simulation studies. The empirical analyses based on the SHMAS and UK Biobank datasets further demonstrate the applicability of the method. Exploratory analyses suggest that the modeling assumptions, including the use of a continuous response and identity link, provide a reasonable approximation for the observed data. While the focus of this work is methodological, these examples illustrate how the proposed approach can be applied in realistic settings with correlated observations.

The proposed method naturally accommodates the use of working correlation structures, as generalized estimating equation (GEE) estimators remain consistent even when the working covariance structure is misspecified. To further reduce computational burden, variance-related nuisance parameters may be estimated using only a subset of observation times, depending on the underlying longitudinal time pattern. In this study, correlation parameters were estimated using Pearson residuals; more sophisticated approaches for correlation estimation have been developed in the literature [39, 40] and can be readily incorporated into our framework.

Several limitations should be noted. First, the theoretical results are developed under the standard GEE asymptotic framework where the number of clusters *n* diverges and cluster sizes remain bounded, and may not directly apply to settings with a small number of clusters and large within-cluster observations. When the number of observations per cluster is potentially infinite, as in time series settings, alternative block perturbation schemes–analogous to block bootstrap methods such as the moving block bootstrap and stationary bootstrap [41–44] may be employed. Second, the proposed method inherits the robustness properties of GEE with respect to correlation misspecification, but like standard GEE estimators, it may be sensitive to extreme outliers. Third, the real-data analysis relies on complete-case observations, which assumes that missingness is independent of the outcome conditional on observed covariates. This assumption may not hold in practice, and extending the proposed framework to accommodate informative missingness would be an important direction for future work.

## Appendix A Theoretical properties

In this section, we study the theoretical properties of the resulting estimators obtained from the above Algorithm 1. Note that the stochastic weights $W_{n}$ in the algorithm are independent of the data $(X_{it}, Y_{it}), i=1,\ldots , n, t=1, \ldots , t_0$. Condition on data, only the stochastic weight is random while unconditionally, the randomness of the resulting estimator involves both the stochastic weights and data. We will use $Pr^*$, $E^*$ and $\text {var}^*$ to denote conditional perturbation probability, conditional expectation and conditional variance given data respectively. We assume $r_n, m<n$ and the following regularity conditions.

### Assumption 1

The parameter space Θ is compact in $\mathbb {R}^p$ and the true value $\boldsymbol{\beta }_0$ is an interior point.

### Assumption 2

Suppose that for each fixed nuisance parameter $\alpha , \phi$, the general estimating function admits an objective function $l (\boldsymbol{\beta },Y)$ such that $\frac{\partial l}{\partial \boldsymbol{\beta }} (\boldsymbol{\beta },Y) = {U} (\boldsymbol{\beta },Y)$ where $|l (\boldsymbol{\beta },\cdot )| < L (\cdot )$ for all $\boldsymbol{\beta } \in \Theta$ and that $L (\cdot ) < \infty$. Similarly, the subsample stochastic estimating function $\tilde{U} (\boldsymbol{\beta },Y,W_n)$ admits a corresponding objective function $\tilde{l} (\boldsymbol{\beta },Y,W_n)$ such that $\frac{\partial \tilde{l}}{\partial \boldsymbol{\beta }} (\boldsymbol{\beta },Y,W_n) = \tilde{U} (\boldsymbol{\beta },Y,W_n)$ both conditionally on the observed data Y=y and unconditionally. Further, assume that there exists measures ν and ω such that the normalizing integral of the exponential objective function is finite and does not depend on $\boldsymbol{\beta }$, that is

$$\begin{aligned} \int \int e^{\tilde{l} (\boldsymbol{\beta },y,W_n)} d\nu (y)d\omega (w) = c. \end{aligned}$$

for some constant *c* independent of $\boldsymbol{\beta }$.

### Assumption 3

Assume that the nuisance parameter estimates satisfy: (1) $\tilde{\alpha }_{r_n}$ is $r_n^{1/2}$ consistent given $\boldsymbol{\beta }$ and ϕ, (2) $\tilde{\phi }_{r_n}$ is $r_n^{1/2}$ consistent given $\boldsymbol{\beta }$, (3) $\vert \partial \tilde{\alpha }_{r_n} (\boldsymbol{\beta }, \phi )/\partial \phi \vert \le H (\boldsymbol{\beta })$ and $H (\cdot ) < \infty$, and that (4) $\lim \sup _{n \rightarrow \infty }E(\partial U/\partial \alpha )^2 < \infty$.

### Assumption 4

$\partial U/\partial \boldsymbol{\beta }$ exists for all $\boldsymbol{\beta } \in \Theta$ and $\lim \sup _{n \rightarrow \infty }E(\partial U/\partial \boldsymbol{\beta })^2 < \infty$.

### Assumption 5

Assume that $B_{n,i} \sim \texttt {Bernoulli} (q_{n,i})$, $q_{n,i} \in (0,1]$ such that $\sum _{i=1}^n q_{n,i} = r_n$, $E (G_{n,i})=\frac{1}{q_{n,i}}$ and $\text {var}(G_{n,i}) = b_{n,i}^2$ and there exists α>0 such that $\limsup _{n \rightarrow \infty } (r_n/n)^{1+\alpha } EG_{n,i}^{2+\alpha } < \infty$ for i=1,…,n.

Assumption 1-2 are required to guarantee consistency of solutions of estimating equations. Assumption 3 imposes assumption to nuisance parameters. Assumption 4 is used in Taylor expansion and Lindeberg condition for central limit theorem [45]. Assumption 5 is on the requirement of the stochastic weights.

### Remark A.1

Assumption 5 imposes moment conditions on the stochastic weights to facilitate asymptotic normality. While the condition is stated in a general form, it is mild and satisfied by commonly used choices such as Gamma, Beta, and Geometric weights described in Remark 1. In particular, finite second moments are sufficient for most practical implementations. The higher-order moment condition is only used to justify a central limit theorem for weighted estimating equations and does not impose restrictive requirements in typical applications. To improve clarity, we have avoided emphasizing technical conditions such as the Lindeberg condition in the main text.

Our first theorem establishes the consistency of the estimator $\tilde{\boldsymbol{\beta }}_{n,k}, k=1, \ldots , m$, under mild conditions. The result holds as both $r_n \rightarrow \infty$ and $n \rightarrow \infty$, with $r_n/n \rightarrow 0$, which corresponds to the subsampling regime considered in large-scale data settings. Importantly, consistency does not rely on the higher-order moment conditions required for asymptotic normality.

### Theorem A.1

Under the Assumption 1 and 2, the global maximum of $\tilde{l} (\boldsymbol{\beta },y,W_n)$ is a consistent estimate of full data GEE estimator $\hat{\boldsymbol{\beta }}_n$ condition on the data, and the global maximum of $\tilde{l} (\boldsymbol{\beta },Y,W_n)$ is a consistent estimate of $\boldsymbol{\beta }_0$ as $r_n, n \rightarrow \infty$.

The second theorem establishes the asymptotic conditional normality of estimator (7) in Algorithm 1.

### Theorem A.2

Condition on data, under Assumptions 1-5, $(r_nm)^{1/2} (\tilde{\boldsymbol{\beta }}^{ (m)}_n - \hat{\boldsymbol{\beta }}_n)$ is asymptotically multivariate Gaussian with zero mean and variance-covariance matrix given by

A1 $$\begin{aligned} \begin{aligned} \lim _{n \rightarrow \infty } r_n&\left( \sum _{i=1}^n (Q_i (\hat{\boldsymbol{\beta }}_n) + D_i (\hat{\boldsymbol{\beta }}_n)^TV_i (\hat{\boldsymbol{\beta }}_n)^{-1}D_i (\hat{\boldsymbol{\beta }}_n) )\right) ^{-1}\\ \times&\left( \sum _{i=1}^n (\frac{1}{q_{n,i}}-1+b_{n,i}^2q_{n,i}) D_i (\hat{\boldsymbol{\beta }}_n)^T V_i (\hat{\boldsymbol{\beta }}_n)^{-1} (Y_i - \mu _i(\hat{\boldsymbol{\beta }}_n) )^2V_i (\hat{\boldsymbol{\beta }}_n)^{-1}D_i (\hat{\boldsymbol{\beta }}_n) \right) \\ \times&\left( \sum _{i=1}^n (Q_i (\hat{\boldsymbol{\beta }}_n) + D_i (\hat{\boldsymbol{\beta }}_n)^TV_i (\hat{\boldsymbol{\beta }}_n)^{-1}D_i (\hat{\boldsymbol{\beta }}_n)) \right) ^{-1} \end{aligned} \end{aligned}$$

as $r_n, n \rightarrow \infty$, and $Q_i$ is a p×p matrix given by

$$\begin{aligned} Q_i (\boldsymbol{\beta }) = \frac{\partial D_i (\boldsymbol{\beta }) V_i^{-1} (\boldsymbol{\beta })}{\partial \boldsymbol{\beta }^T} (Y_i-\mu _i({\boldsymbol{\beta }})). \end{aligned}$$

### Remark A.2

If the Poisson sampling reduces to the Bernoulli sampling in which $q_{n,i} = \frac{r_n}{n}$ and $\text {var}(G_{n,i}) = b_n^2$ for i=1,…,n, then the conditional variance-covariance matrix of $(r_nm)^{1/2} (\tilde{\boldsymbol{\beta }}^{ (m)}_n - \hat{\boldsymbol{\beta }}_n)$ can be written as

A2 $$\begin{aligned} \begin{aligned} \lim _{n \rightarrow \infty } g(r_n,n)&\left( \sum _{i=1}^n (Q_i (\hat{\boldsymbol{\beta }}_n) + D_i (\hat{\boldsymbol{\beta }}_n)^TV_i (\hat{\boldsymbol{\beta }}_n)^{-1}D_i (\hat{\boldsymbol{\beta }}_n) )\right) ^{-1}\\ \times&\left( \sum _{i=1}^n D_i (\hat{\boldsymbol{\beta }}_n)^T V_i (\hat{\boldsymbol{\beta }}_n)^{-1} (Y_i - \mu _i(\hat{\boldsymbol{\beta }}_n) )^2V_i (\hat{\boldsymbol{\beta }}_n)^{-1}D_i (\hat{\boldsymbol{\beta }}_n) \right) \\ \times&\left( \sum _{i=1}^n (Q_i (\hat{\boldsymbol{\beta }}_n) + D_i (\hat{\boldsymbol{\beta }}_n)^TV_i (\hat{\boldsymbol{\beta }}_n)^{-1}D_i (\hat{\boldsymbol{\beta }}_n)) \right) ^{-1} \end{aligned} \end{aligned}$$

where factor $$g(r_n,n) = n-r_n + b_n^2 r_n^2/n$$.

The third theorem establishes the asymptotic unconditional normality of estimator (7).

### Theorem A.3

When $r_n m< n$, under Assumptions 1-5, $(r_nm)^{1/2} (\tilde{\boldsymbol{\beta }}^{ (m)}_n - \boldsymbol{\beta }_0)$ is asymptotically multivariate Gaussian with zero mean and variance-covariance matrix given by

A3 $$\begin{aligned}  &   \lim _{n \rightarrow \infty } r_n m (\sum _{i=1}^n D_i^TV_i^{-1}D_i)^{-1} \left( \sum _{i=1}^n ( 1 + \frac{\frac{1}{q_{n,i}}-1+b_{n,i}^2q_{n,i}}{m} ) D_i^T V_i^{-1} \text {cov}(Y_i) V_i^{-1}D_i \right) \nonumber \\  &   \quad \times (\sum _{i=1}^n D_i^TV_i^{-1}D_i)^{-1} . \end{aligned}$$

as $r_n, n \rightarrow \infty$. When $r_nm \ge n$, under Assumption 1-5, $n^{1/2} (\tilde{\boldsymbol{\beta }}^{ (m)}_n - \boldsymbol{\beta }_0)$ is asymptotically multivariate Gaussian with zero mean and variance-covariance matrix given by

A4 $$\begin{aligned}  &   \lim _{n \rightarrow \infty } n (\sum _{i=1}^n D_i^TV_i^{-1}D_i)^{-1} \left( \sum _{i=1}^n ( 1 + \frac{\frac{1}{q_{n,i}}-1+b_{n,i}^2q_{n,i}}{m} ) D_i^T V_i^{-1} \text {cov} (Y_i) V_i^{-1}D_i \right) \nonumber \\  &   \quad \times (\sum _{i=1}^n D_i^TV_i^{-1}D_i)^{-1}. \end{aligned}$$

### Remark A.3

If the Poisson sampling reduces to the Bernoulli sampling in which $q_{n,i} = \frac{r_n}{n}$ and $\text {var}(G_{n,i}) = b_n^2$ for i=1,…,n, when $r_n m<n$, the unconditional variance-covariance matrix of $(r_nm)^{1/2} (\tilde{\boldsymbol{\beta }}^{ (m)}_n - \boldsymbol{\beta }_0)$ can be written as

A5 $$\begin{aligned} \begin{aligned} \lim _{n \rightarrow \infty } s(m, r_n,n) (\sum _{i=1}^n D_i^TV_i^{-1}D_i)^{-1} \left( \sum _{i=1}^n D_i^T V_i^{-1} \text {cov} (Y_i) V_i^{-1}D_i \right) (\sum _{i=1}^n D_i^TV_i^{-1}D_i)^{-1} \end{aligned} \end{aligned}$$

where factor $s(m, r_n,n) = n -r_n + b_n^2 r_n^2/n + mr_n$. When $r_nm\ge n$, the unconditional variance-covariance matrix of $n^{1/2} (\tilde{\boldsymbol{\beta }}^{ (m)}_n - \boldsymbol{\beta }_0)$ can be written as

A6 $$\begin{aligned} \begin{aligned} \lim _{n \rightarrow \infty } t(m, r_n,n) (\sum _{i=1}^n D_i^TV_i^{-1}D_i)^{-1} \left( \sum _{i=1}^n D_i^T V_i^{-1} \text {cov} (Y_i) V_i^{-1}D_i \right) (\sum _{i=1}^n D_i^TV_i^{-1}D_i)^{-1} \end{aligned} \end{aligned}$$

where $t(m, r_n,n) = n+\frac{n^2}{m r_n} (1-r_n/n+b_n^2r_n^2/n^2)$. Hence, the unconditional variance-covariance matrix of $\tilde{\boldsymbol{\beta }}_{n}^{(m)}$ can then be approximated by $h(m,r_n,n) = \frac{r_n m}{n-r_n + b_n^2 r_n^2/n} +1$ times the corresponding conditional variance-covariance matrix, which can be obtained by formula (8) in practice.

### Remark A.4

The unconditional variance-covariance matrix of the perturbation estimator is given by $s(m, r_n,n)/(r_nm) = t(m, r_n,n)/n = 1 + \frac{1}{m}(\frac{n}{r_n}-1+\frac{b_n^2 r_n}{n}) (> 1)$ times the variance of the estimator from the full data. Different choices of subsample size, perturbation time, stochastic weighting random variable will yield different coefficients of the asymptotic variance. We can use the unconditional variance of repeated block perturbation estimator to estimate the variance of full data estimator, which might be computationally unavailable when sample size is extremely large.

## Appendix B Proof of Theorem A.1

Notice that

B7 $$\begin{aligned} \begin{aligned} \tilde{l} (\boldsymbol{\beta },y,W_n) = \frac{1}{n}\sum _{i=1}^n W_{n,i} l (\boldsymbol{\beta },y_i). \end{aligned} \end{aligned}$$

For any $\boldsymbol{\beta } \in \Theta$, we have:

$$\begin{aligned} {E}^* (\tilde{l} (\boldsymbol{\beta },y,W_n)) = l (\boldsymbol{\beta },y), \end{aligned}$$

and based on (B7)

$$\begin{aligned} \text {var}^*(\tilde{l} (\boldsymbol{\beta },y,W_n)) = \frac{1}{n}\sum _{i=1}^n \left( \frac{1}{n}\text {var} (W_{n,i}) \right) l^2 (\boldsymbol{\beta },y_i) \le \left( \frac{1}{n}\max _{i\le n}\text {var} (W_{n,i}) \right) \frac{1}{n}\sum _{i=1}^n l^2 (\boldsymbol{\beta },y_i). \end{aligned}$$

Given that $\lim \sup _{n\rightarrow \infty }\text {var} (W_{n,i})/\frac{n}{r_n} < \infty$ for i=1,…,n, the conditional variance of $\tilde{l} (\boldsymbol{\beta },y,W_n)$ is then satisfying that $\lim \sup _{ n\rightarrow \infty }\text {var}^* (\tilde{l} (\boldsymbol{\beta },y,W_n))/\frac{1}{r_n} < \infty$. By the Markov inequality, for every ϵ>0,

$$\begin{aligned} Pr^* (|\tilde{l} (\boldsymbol{\beta },y,W_n)-l (\boldsymbol{\beta },y)| \ge \epsilon ) \le \frac{\text {var}^*(\tilde{l} (\boldsymbol{\beta },y,W_n)) }{\epsilon ^2} \rightarrow 0 ~\text{ as } n,r_n \rightarrow \infty . \end{aligned}$$

Therefore, $\tilde{l} (\boldsymbol{\beta },y,W_n)$ converges weakly to the $l (\boldsymbol{\beta },y)$ condition on the data. To establish the uniform convergence in probability, we observe that $l (\boldsymbol{\beta },\cdot )<L (\cdot )$ for all $\boldsymbol{\beta } \in \Theta$ under Assumption 2. Since $E^* (W_nL (\cdot )) = L (\cdot ) < \infty$ , we have

$$\begin{aligned} Pr^* (\sup _{\boldsymbol{\beta }\in \Theta }|\tilde{l} (\boldsymbol{\beta },y,W_n)-l (\boldsymbol{\beta },y)| \ge \epsilon ) \rightarrow 0 \end{aligned}$$

as $r_n, n \rightarrow \infty$. Given that $\hat{\boldsymbol{\beta }}_n$ is the maximizer of the $l (\boldsymbol{\beta },y)$ and $\tilde{\boldsymbol{\beta }}_n$ is maximizer of $\tilde{l} (\boldsymbol{\beta },y,W_n)$ by definition condition on the data, the conditional consistency of the maximizer can be established by adapting the Argmax theorem under Assumption 1 [46].

Unconditionally, we need to first show that the expectation $E\tilde{l} (\boldsymbol{\beta },Y,W_n)$ is maximized at $\boldsymbol{\beta }_0$. The argument is similar in spirit to the Proposition 1 in [47]. For each $\boldsymbol{\beta } \in \Theta$ and each fixed nuisance parameter $\alpha , \phi$, define a probability measure

$$\begin{aligned} d\tilde{Q}_{\boldsymbol{\beta }} (y,W_n) = e^{\tilde{l} (\boldsymbol{\beta }, y,W_n)}d\nu (y)d\omega (w)/c. \end{aligned}$$

Differentiating the equation in the proposition with respect to $\boldsymbol{\beta }$, we have $\mu (\boldsymbol{\beta }) = \int y d\tilde{Q}_{\boldsymbol{\beta }} (y,W_n)$. Since $\tilde{l} (\boldsymbol{\beta },Y,W_n)$ is a linear function of *Y*, it follows that $E\tilde{l} (\boldsymbol{\beta },Y,W_n) = \int \tilde{l} (\boldsymbol{\beta },y,W_n)d\tilde{Q}_{\boldsymbol{\beta }_0} (y,W_n)$ for all $\boldsymbol{\beta }$. Hence, by Jensen’s inequality,

$$\begin{aligned} E (\tilde{l} (\boldsymbol{\beta },Y,W_n) - \tilde{l} (\boldsymbol{\beta }_0,Y,W_n))&= \int \{\tilde{l} (\boldsymbol{\beta },y,W_n) - \tilde{l} (\boldsymbol{\beta }_0,y,W_n)\}d\tilde{Q}_{\boldsymbol{\beta }_0} (y,W_n)\\&< \log \int e^{\tilde{l} (\boldsymbol{\beta },y,W_n) - \tilde{l} (\boldsymbol{\beta }_0,y,W_n)} d\tilde{Q}_{\boldsymbol{\beta }_0} (y,W_n) \\&= \log \int \{d\tilde{Q}_{\boldsymbol{\beta }} (y,W_n)/d\tilde{Q}_{\boldsymbol{\beta }_0} (y,W_n)\}d\tilde{Q}_{\boldsymbol{\beta }_0} (y,W_n) = 0. \end{aligned}$$

That is, the expectation the expectation $E\tilde{l} (\boldsymbol{\beta },Y,W_n)$ is maximized at $\boldsymbol{\beta }_0$. For any $\boldsymbol{\beta } \in \Theta$, we can calculate the variance based on (B7)

$$\begin{aligned} \text {var} (\tilde{l} (\boldsymbol{\beta },Y,W_n)) =&\text {var} (\frac{1}{n}\sum _{i=1}^n W_{n,i} l (\boldsymbol{\beta },Y)) \\ =&\left( \frac{1}{n^2} \sum _{i=1}^n \text {var} (W_{n,i}) \right) (El (\boldsymbol{\beta },Y))^2 + \left( \frac{1}{n^2} \sum _{i=1}^n \text {E} (W_{n,i})^2 \right) \text {var} (l (\boldsymbol{\beta },Y)). \end{aligned}$$

Given that $\lim \sup _{n\rightarrow \infty }\text {E} (W_{n,i})^2/\frac{n}{r_n} < \infty$ and $\text {E} (W_{n,i}) =1$ for i=1,…,n, the unconditional variance of $\tilde{l} (\boldsymbol{\beta },Y,W_n)$ is satisfying that $\lim \sup _{ n\rightarrow \infty }\text {var} (\tilde{l} (\boldsymbol{\beta },Y,W_n))/\frac{1}{r_n} < \infty$. By the Markov’s inequality and the bounded condition of l(·) imposed by Assumption 2, the convergence in probability of $\tilde{l} (\boldsymbol{\beta },Y,W_n)$ to $E\tilde{l} (\boldsymbol{\beta },Y,W_n)$ uniformly in $\boldsymbol{\beta } \in \Theta$ is ensured. We then have the consistency of the maximizers by adapting the Argmax theorem under Assumption 1 [46]. This completes the proof.

## Appendix C Proof of Theorem A.2

We use $\tilde{\boldsymbol{\beta }}_n$ as the general notation for the single perturbation estimator. Condition on the data, $\hat{\boldsymbol{\beta }}_n$ is fixed. Do Taylor expansion of $\sum _{i=1}^n W_{n,i}U_i \left( \boldsymbol{\beta }, \tilde{\alpha }_{r_n} (\boldsymbol{\beta }, \tilde{\phi }_{r_n} ( \boldsymbol{\beta })) \right)$ around $\hat{\boldsymbol{\beta }}_n$,

$$\begin{aligned} 0&= \sum _{i=1}^n W_{n,i} D_i (\tilde{\boldsymbol{\beta }}_n) V_i^{-1} (\tilde{\alpha }_{r_n} (\tilde{\boldsymbol{\beta }}_n, \tilde{\phi }_{r_n} (\tilde{\boldsymbol{\beta }}_n)) (Y_i - \mu _i(\tilde{\boldsymbol{\beta }}_n)) \\&= \sum _{i=1}^n W_{n,i} D_i (\hat{\boldsymbol{\beta }}_n) V_i^{-1} (\tilde{\alpha }_{r_n} (\hat{\boldsymbol{\beta }}_n, \tilde{\phi }_{r_n} (\hat{\boldsymbol{\beta }}_n)) (Y_i - \mu _i(\hat{\boldsymbol{\beta }}_n) ) \\&\quad + \sum _{i=1}^n W_{n,i} (\tilde{\boldsymbol{\beta }}_n- \hat{\boldsymbol{\beta }}_n) \left( \tilde{Q}_i (\boldsymbol{\beta }^*) - D_i (\boldsymbol{\beta }^*) V_i^{-1} (\tilde{\alpha }_{r_n} (\boldsymbol{\beta }^*, \tilde{\phi }_{r_n} ( \boldsymbol{\beta }^*))D_i (\boldsymbol{\beta }^*) \right) \end{aligned}$$

where $\tilde{Q}_i$ is a p×p matrix given by

$$\begin{aligned} \tilde{Q}_i (\boldsymbol{\beta }) = \frac{\partial D_i (\boldsymbol{\beta }) V_i^{-1} (\tilde{\alpha }_{r_n} (\boldsymbol{\beta }, \tilde{\phi }_{r_n} (\boldsymbol{\beta })) }{\partial \boldsymbol{\beta }^T} (Y_i-\mu _i({\boldsymbol{\beta }})), \end{aligned}$$

and $\boldsymbol{\beta }^*$ is some value between $\tilde{\boldsymbol{\beta }}_n$ and $\hat{\boldsymbol{\beta }}_n$. Then we obtain:

C8 $$\begin{aligned} \begin{aligned} r_n^{1/2} (\tilde{\boldsymbol{\beta }}_n - \hat{\boldsymbol{\beta }}_n) =&\left( \frac{1}{n} \sum _{i=1}^n W_{n,i} \left( -\tilde{Q}_i (\boldsymbol{\beta }^*) + D_i (\boldsymbol{\beta }^*) V_i^{-1} (\tilde{\alpha }_{r_n} (\boldsymbol{\beta }^*, \tilde{\phi }_{r_n} ( \boldsymbol{\beta }^*))D_i (\boldsymbol{\beta }^*) \right) \right) ^{-1}\\&\times \frac{r_n^{1/2}}{n} \left( \sum _{i=1}^n W_{n,i} D_i (\hat{\boldsymbol{\beta }}_n) V_i^{-1} (\tilde{\alpha }_{r_n} (\hat{\boldsymbol{\beta }}_n, \tilde{\phi }_{r_n} ( \hat{\boldsymbol{\beta }}_n)) (Y_i - \mu _i(\hat{\boldsymbol{\beta }}_n) ) \right) \\ =&\left( \frac{1}{n} \sum _{i=1}^n A_i + \frac{1}{n} \sum _{i=1}^n B_i \right) \times \frac{r_n^{1/2}}{n} \left( \sum _{i=1}^n W_{n,i} U_i \left( \hat{\boldsymbol{\beta }}_n, \tilde{\alpha }_{r_n} (\hat{\boldsymbol{\beta }}_n, \tilde{\phi }_{r_n} ( \hat{\boldsymbol{\beta }}_n )) \right) \right) \end{aligned} \end{aligned}$$

where $A_i = W_{n,i} \tilde{Q}_i(\boldsymbol{\beta }^*)$, $B_i = W_{n,i}D_i (\boldsymbol{\beta }^*) V_i^{-1} (\tilde{\alpha }_{r_n} (\boldsymbol{\beta }^*, \tilde{\phi }_{r_n} ( \boldsymbol{\beta }^*))D_i (\boldsymbol{\beta }^*)$ and we denote the estimating function $U_i \left( \hat{\boldsymbol{\beta }}_n, \tilde{\alpha }_{r_n} (\hat{\boldsymbol{\beta }}_n, \tilde{\phi }_{r_n} ( \hat{\boldsymbol{\beta }}_n ) ) \right) = D_i (\hat{\boldsymbol{\beta }}_n) V_i^{-1} (\tilde{\alpha }_{r_n} (\hat{\boldsymbol{\beta }}_n, \tilde{\phi }_{r_n} ( \hat{\boldsymbol{\beta }}_n)) (Y_i - \mu _i(\hat{\boldsymbol{\beta }}_n) )$.

Notice that

$$\begin{aligned} \frac{1}{n} \sum _{i=1}^n A_i =&\left( \frac{1}{n} \sum _{i=1}^n W_{n,i} \tilde{Q}_i (\boldsymbol{\beta }^*) - \frac{1}{n} \sum _{i=1}^n \tilde{Q}_i (\boldsymbol{\beta }^*) \right) \\&+ \left( \frac{1}{n} \sum _{i=1}^n \tilde{Q}_i (\boldsymbol{\beta }^*) - \frac{1}{n} \sum _{i=1}^n \tilde{Q}_i (\hat{\boldsymbol{\beta }}_n) \right) + \frac{1}{n} \sum _{i=1}^n \tilde{Q}_i (\hat{\boldsymbol{\beta }}_n). \end{aligned}$$

By Assumption 4 and $\lim \sup _{n\rightarrow \infty }\text {var} (W_{n,i})/\frac{n}{r_n} < \infty$, $\limsup _{n\rightarrow \infty }\text {var}^* (\frac{1}{n} \sum _{i=1}^n W_{n,i} \tilde{Q}_i (\boldsymbol{\beta }^*))/\frac{1}{r_n}<\infty$. Since $E^* (\frac{1}{n} \sum _{i=1}^n W_{n,i} \tilde{Q}_i (\boldsymbol{\beta }^*)) = \frac{1}{n} \sum _{i=1}^n \tilde{Q}_i (\boldsymbol{\beta }^*)$, then $\left( \frac{1}{n} \sum _{i=1}^n W_{n,i} \tilde{Q}_i (\boldsymbol{\beta }^*) - \frac{1}{n} \sum _{i=1}^n \tilde{Q}_i (\boldsymbol{\beta }^*) \right) = o_{p^*} (1)$ by Markov’s inequality. By the the consistency between $\tilde{\boldsymbol{\beta }}_n$ and $\hat{\boldsymbol{\beta }}_n$,

$\left( \frac{1}{n} \sum _{i=1}^n \tilde{Q}_i (\boldsymbol{\beta }^*) - \frac{1}{n} \sum _{i=1}^n \tilde{Q}_i (\hat{\boldsymbol{\beta }}_n) \right) = o_{p^*} (1)$ by continuous mapping theorem. Therefore,

C9 $$\begin{aligned} \frac{1}{n} \sum _{i=1}^n A_i = \frac{1}{n} \sum _{i=1}^n \tilde{Q}_i (\hat{\boldsymbol{\beta }}_n) + o_{p^*} (1). \end{aligned}$$

Next we investigate the consistency of the nuisance parameters. Note that

C10 $$\begin{aligned} V_i^{-1} (\tilde{\alpha }_{r_n} (\hat{\boldsymbol{\beta }}_n, \tilde{\phi }_{r_n} ( \hat{\boldsymbol{\beta }}_n)) = V_i^{-1} ({\alpha } (\hat{\boldsymbol{\beta }}_n, {\phi } ( \hat{\boldsymbol{\beta }}_n)) + r_n^{-1/2} \frac{\partial }{\partial \alpha } \left( V_i^{-1} ({\alpha } (\hat{\boldsymbol{\beta }}_n, {\phi } ( \hat{\boldsymbol{\beta }}_n)) \right) r_n^{1/2} (\alpha ^*-\alpha ) \end{aligned}$$

where $\alpha ^*$ is between $\tilde{\alpha }_{r_n}$ and α and

$$\begin{aligned} r_n^{1/2} (\alpha ^*-\alpha ) =&r_n^{1/2} (\alpha ^* (\hat{\boldsymbol{\beta }}_n, {\phi }^* ( \hat{\boldsymbol{\beta }}_n))-\alpha )\\ =&r_n^{1/2} (\alpha ^* (\hat{\boldsymbol{\beta }}_n, {\phi }^* (\hat{\boldsymbol{\beta }}_n))-\alpha ^* ( \hat{\boldsymbol{\beta }}_n, {\phi } ( \hat{\boldsymbol{\beta }}_n)) + \alpha ^* ( \hat{\boldsymbol{\beta }}_n, {\phi } ( \hat{\boldsymbol{\beta }}_n)) - \alpha )\\ =&r_n^{1/2}\left( \frac{\partial \alpha ^*}{\partial \phi } (\hat{\boldsymbol{\beta }}_n, {\phi }^{**}) ({\phi }^*-\phi ) \right) + r_n^{1/2}\left( \alpha ^* ( \hat{\boldsymbol{\beta }}_n, {\phi } (\hat{\boldsymbol{\beta }}_n)) - \alpha \right) = O_{p^*} (1) \end{aligned}$$

by the consistency of $\tilde{\alpha }_{r_n}$ and $\tilde{\phi }_{r_n}$ under Assumption 3. Hence under Assumption 4, we have

$$V_i^{-1} (\tilde{\alpha }_{r_n} (\hat{\boldsymbol{\beta }}_n, \tilde{\phi }_{r_n} ( \hat{\boldsymbol{\beta }}_n)) = V_i^{-1} ({\alpha } (\hat{\boldsymbol{\beta }}_n, {\phi } ( \hat{\boldsymbol{\beta }}_n)) + O_{p^*} (r_n^{-1/2})$$, and therefore

C11 $$\begin{aligned} \frac{1}{n} \sum _{i=1}^n \tilde{Q}_i (\hat{\boldsymbol{\beta }}_n) = \frac{1}{n} \sum _{i=1}^n Q_i (\hat{\boldsymbol{\beta }}_n) + o_{p^*} (1). \end{aligned}$$

where $Q_i$ is a p×p matrix given by

$$\begin{aligned} Q_i (\boldsymbol{\beta }) = \frac{\partial D_i (\boldsymbol{\beta }) V_i^{-1} (\boldsymbol{\beta })}{\partial \boldsymbol{\beta }^T} (Y_i-\mu _i({\boldsymbol{\beta }}) ). \end{aligned}$$

Combine (C9) and (C11), we have

C12 $$\begin{aligned} \frac{1}{n} \sum _{i=1}^n A_i = \frac{1}{n} \sum _{i=1}^n Q_i (\hat{\boldsymbol{\beta }}_n) + o_{p^*} (1). \end{aligned}$$

Similarly, we have

C13 $$\begin{aligned} \frac{1}{n} \sum _{i=1}^n B_i = \frac{1}{n} \sum _{i=1}^n D_i (\hat{\boldsymbol{\beta }}_n) V_i^{-1} (\tilde{\alpha }_{r_n} (\hat{\boldsymbol{\beta }}_n, \tilde{\phi }_{r_n} ( \hat{\boldsymbol{\beta }}_n))D_i (\hat{\boldsymbol{\beta }}_n) + o_{p^*} (1) \end{aligned}$$

and that

C14 $$\begin{aligned} \frac{1}{n} \sum _{i=1}^n D_i (\hat{\boldsymbol{\beta }}_n) V_i^{-1} (\tilde{\alpha }_{r_n} (\hat{\boldsymbol{\beta }}_n, \tilde{\phi }_{r_n} ( \hat{\boldsymbol{\beta }}_n))D_i (\hat{\boldsymbol{\beta }}_n) = \frac{1}{n} \sum _{i=1}^n D_i (\hat{\boldsymbol{\beta }}_n) V_i^{-1} ({\alpha } (\hat{\boldsymbol{\beta }}_n, {\phi } (\hat{\boldsymbol{\beta }}_n))D_i (\hat{\boldsymbol{\beta }}_n) + o_{p^*} (1). \end{aligned}$$

Combine (C13) and (C14), we have

C15 $$\begin{aligned} \frac{1}{n} \sum _{i=1}^n B_i = \frac{1}{n} \sum _{i=1}^n D_i (\hat{\boldsymbol{\beta }}_n) V_i^{-1} ({\alpha } (\hat{\boldsymbol{\beta }}_n, {\phi } (\hat{\boldsymbol{\beta }}_n))D_i (\hat{\boldsymbol{\beta }}_n) + o_{p^*} (1). \end{aligned}$$

Therefore, by (C12) and (C15),

C16 $$\begin{aligned} \frac{1}{n} \sum _{i=1}^n A_i + \frac{1}{n} \sum _{i=1}^n B_i = \frac{1}{n} \sum _{i=1}^n Q_i (\hat{\boldsymbol{\beta }}_n) + \frac{1}{n} \sum _{i=1}^n D_i (\hat{\boldsymbol{\beta }}_n) V_i^{-1} ({\alpha } (\hat{\boldsymbol{\beta }}_n, {\phi } (\hat{\boldsymbol{\beta }}_n))D_i (\hat{\boldsymbol{\beta }}_n) + o_{p^*} (1). \end{aligned}$$

Given that $\tilde{\boldsymbol{\beta }}_n^{ (m)} = \frac{1}{m} \sum _{k=1}^m \tilde{\boldsymbol{\beta }}_{n,k}$ and equation (C8), (C16)

C17 $$\begin{aligned} \begin{aligned} (r_n m )^{1/2} (\tilde{\boldsymbol{\beta }}_n^{ (m)} - \hat{\boldsymbol{\beta }}_n)&= m^{-1/2} \sum _{k=1}^m \left( r_n^{1/2} (\tilde{\boldsymbol{\beta }}_{n,k} - \hat{\boldsymbol{\beta }}_n) \right) \\&= \left( \frac{1}{n} \sum _{i=1}^n Q_i (\hat{\boldsymbol{\beta }}_n) + \frac{1}{n} \sum _{i=1}^n D_i (\hat{\boldsymbol{\beta }}_n) V_i^{-1} ({\alpha } (\hat{\boldsymbol{\beta }}_n, {\phi } (\hat{\boldsymbol{\beta }}_n))D_i (\hat{\boldsymbol{\beta }}_n) \right) ^{-1}\\&\quad \times \sum _{i=1}^n \frac{r_n^{1/2}}{nm^{1/2}} \left( \sum _{k=1}^m W_{n,i,k} U_i \left( \hat{\boldsymbol{\beta }}_n, \tilde{\alpha }_{r_n} (\hat{\boldsymbol{\beta }}_n, \tilde{\phi }_{r_n} ( \hat{\boldsymbol{\beta }}_n ) ) \right) \right) \\&\quad + o_{p^*}\left( \sum _{i=1}^n \frac{r_n^{1/2}}{nm^{1/2}} \left( \sum _{k=1}^m W_{n,i,k} U_i \left( \hat{\boldsymbol{\beta }}_n, \tilde{\alpha }_{r_n} (\hat{\boldsymbol{\beta }}_n, \tilde{\phi }_{r_n} ( \hat{\boldsymbol{\beta }}_n ) ) \right) \right) \right) , \end{aligned} \end{aligned}$$

where $W_{n,i,k}$ represents the stochastic weight assigned to the *i*th objective function in *k*th perturbation. Now we show that $\sum _{i=1}^n \frac{r_n^{1/2}}{nm^{1/2}} ( \sum _{k=1}^m W_{n,i,k}) U_i \left( \hat{\boldsymbol{\beta }}_n, \tilde{\alpha }_{r_n} (\hat{\boldsymbol{\beta }}_n, \tilde{\phi }_{r_n}\right. \left. ( \hat{\boldsymbol{\beta }}_n ) ) \right) =O_{p^*}(1)$. Given (C10), we have

C18 $$\begin{aligned} \begin{aligned}&\sum _{i=1}^n \frac{r_n^{1/2}}{nm^{1/2}} \left( \sum _{k=1}^m W_{n,i,k} U_i \left( \hat{\boldsymbol{\beta }}_n, \tilde{\alpha }_{r_n} (\hat{\boldsymbol{\beta }}_n, \tilde{\phi }_{r_n} ( \hat{\boldsymbol{\beta }}_n ) ) \right) \right) \\&\quad = \frac{r_n^{1/2}}{nm^{1/2}} \left( \sum _{i=1}^n (\sum _{k=1}^m W_{n,i,k}) D_i (\hat{\boldsymbol{\beta }}_n) V_i^{-1} ({\alpha } (\hat{\boldsymbol{\beta }}_n, {\phi } (\hat{\boldsymbol{\beta }}_n)) (Y_i - \mu _i(\hat{\boldsymbol{\beta }}_n) ) \right) \\&\qquad + r_n^{1/2} (\alpha ^*-\alpha ) \frac{1}{nm^{1/2}}\sum _{i=1}^n (\sum _{k=1}^m W_{n,i,k}) \frac{\partial }{\partial \alpha } \left( D_i (\hat{\boldsymbol{\beta }}_n) V_i^{-1} ({\alpha } (\hat{\boldsymbol{\beta }}_n, {\phi } ( \hat{\boldsymbol{\beta }}_n)) (Y_i - \mu _i(\hat{\boldsymbol{\beta }}_n)) \right) . \end{aligned} \end{aligned}$$

Write $U_i \left( \hat{\boldsymbol{\beta }}_n, {\alpha } (\hat{\boldsymbol{\beta }}_n, {\phi } (\hat{\boldsymbol{\beta }}_n)) \right) = D_i (\hat{\boldsymbol{\beta }}_n) V_i^{-1} ({\alpha } (\hat{\boldsymbol{\beta }}_n, {\phi } ( \hat{\boldsymbol{\beta }}_n)) (Y_i - \mu _i(\hat{\boldsymbol{\beta }}_n))$. Notice that

$$\begin{aligned}&E^*\left( \frac{1}{nm^{1/2}}\sum _{i=1}^n (\sum _{k=1}^m W_{n,i,k}) \frac{\partial }{\partial \alpha } U_i \left( \hat{\boldsymbol{\beta }}_n, {\alpha } (\hat{\boldsymbol{\beta }}_n, {\phi } (\hat{\boldsymbol{\beta }}_n)) \right) \right) \\ =&\frac{m^{1/2}}{n}\sum _{i=1}^n \frac{\partial }{\partial \alpha } U_i \left( \hat{\boldsymbol{\beta }}_n, {\alpha } (\hat{\boldsymbol{\beta }}_n, {\phi } (\hat{\boldsymbol{\beta }}_n)) \right) \\ =&\frac{\partial }{\partial \alpha } \left( \frac{m^{1/2}}{n}\sum _{i=1}^n U_i \left( \hat{\boldsymbol{\beta }}_n, {\alpha } (\hat{\boldsymbol{\beta }}_n, {\phi } (\hat{\boldsymbol{\beta }}_n)) \right) \right) \\ =&\frac{\partial }{\partial \alpha } \left( \frac{m^{1/2}}{n}\sum _{i=1}^n U_i \left( \hat{\boldsymbol{\beta }}_n, \hat{\alpha }_n (\hat{\boldsymbol{\beta }}_n, \hat{\phi }_n (\hat{\boldsymbol{\beta }}_n)) \right) - n^{1/2} (\alpha ^\dagger -\alpha ) \frac{m^{1/2}}{n^{1/2}}\frac{1}{n}\sum _{i=1}^n U_i \left( \hat{\boldsymbol{\beta }}_n, {\alpha } (\hat{\boldsymbol{\beta }}_n, {\phi } (\hat{\boldsymbol{\beta }}_n)) \right) \right) \end{aligned}$$

where $\alpha ^\dagger$ is between $\hat{\alpha }_n$ and α. The interchange of summation and differentiation is by Theorem 7.17 of [48], 3rd Edition, under Assumption 3. Given that $\hat{\boldsymbol{\beta }}_n$ is the solution to the equation $\sum _{i=1}^n U_i \left( {\boldsymbol{\beta }}, \hat{\alpha }_n ({\boldsymbol{\beta }}, \hat{\phi }_n ({\boldsymbol{\beta }})) \right) =0$ we have $\frac{\partial }{\partial \alpha } \left( \frac{m^{1/2}}{n}\sum _{i=1}^n U_i \left( \hat{\boldsymbol{\beta }}_n, \hat{\alpha }_n (\hat{\boldsymbol{\beta }}_n, \hat{\phi }_n (\hat{\boldsymbol{\beta }}_n)) \right) \right) = 0$ and $\hat{\alpha }_n$ and $\hat{\phi }_n$ are $n^{1/2}$-consistent. Hence $n^{1/2} (\alpha ^\dagger -\alpha ) \frac{m^{1/2}}{n^{1/2}}\frac{1}{n}\sum _{i=1}^n U_i \left( \hat{\boldsymbol{\beta }}_n, {\alpha } (\hat{\boldsymbol{\beta }}_n, {\phi } (\hat{\boldsymbol{\beta }}_n)) \right) = o_{p^*}(1)$ when m<n. Therefore the conditional expectation is $o_{p^*}(1)$. Also,

$$\begin{aligned} \text {var}^* \left( \frac{1}{nm^{1/2}}\sum _{i=1}^n (\sum _{k=1}^m W_{n,i,k}) \frac{\partial U_i }{\partial \alpha } \right) = \frac{1}{n^2}\sum _{i=1}^n \text {var}(W_{n,i}) (\frac{\partial U_i }{\partial \alpha })^2, \end{aligned}$$

because $W_{n,i,1}, \ldots , W_{n,i,m}$ are independent and identically distributed condition on the data. It’s easy to see that $\limsup _{n\rightarrow \infty }\text {var}^* \left( \frac{1}{nm^{1/2}}\sum _{i=1}^n (\sum _{k=1}^m W_{n,i,k}) \frac{\partial U_i }{\partial \alpha } \right) /\frac{1}{r_n}<\infty$ given that

$$\lim \sup _{n\rightarrow \infty }\text {var} (W_{n,i})/\frac{n}{r_n} < \infty $$ and Assumption 3.

Hence in (C18), $r_n^{1/2} (\alpha ^*-\alpha ) \frac{1}{nm^{1/2}}\sum _{i=1}^n (\sum _{k=1}^m W_{n,i,k}) \times \frac{\partial }{\partial \alpha } \left( D_i (\hat{\boldsymbol{\beta }}_n) V_i^{-1} ({\alpha } (\hat{\boldsymbol{\beta }}_n, {\phi }\right. \left. ( \hat{\boldsymbol{\beta }}_n)) (Y_i - \mu _i(\hat{\boldsymbol{\beta }}_n)) \right) = o_{p^*}(1)$ and the term

$\sum _{i=1}^n \frac{r_n^{1/2}}{nm^{1/2}} \left( \sum _{k=1}^m W_{n,i,k} U_i \left( \hat{\boldsymbol{\beta }}_n, \tilde{\alpha }_{r_n} (\hat{\boldsymbol{\beta }}_n, \tilde{\phi }_{r_n} ( \hat{\boldsymbol{\beta }}_n ) ) \right) \right)$ is asymptotically equivalent to

C19 $$\begin{aligned} \sum _{i=1}^n \frac{r_n^{1/2}}{nm^{1/2}} (\sum _{k=1}^m W_{n,i,k}) U_i \left( \hat{\boldsymbol{\beta }}_n, {\alpha } (\hat{\boldsymbol{\beta }}_n, {\phi } (\hat{\boldsymbol{\beta }}_n)) \right) , \end{aligned}$$

which is a sum of *n* independent elements, with *i*th term $\frac{r_n^{1/2}}{nm^{1/2}} (\sum _{k=1}^m W_{n,i,k}) U_i \left( \hat{\boldsymbol{\beta }}_n, \right. \left. {\alpha } (\hat{\boldsymbol{\beta }}_n, {\phi } (\hat{\boldsymbol{\beta }}_n)) \right)$.

Define

$$\begin{aligned} {Z}_n^2 =&\left| \left| \sum _{i=1}^n \text {var}^* \left( \frac{r_n^{1/2}}{nm^{1/2}} (\sum _{k=1}^m W_{n,i,k}) U_i \left( \hat{\boldsymbol{\beta }}_n, {\alpha } (\hat{\boldsymbol{\beta }}_n, {\phi } (\hat{\boldsymbol{\beta }}_n)) \right) \right) \right| \right| \\ =&\frac{r_n}{n^2}\sum _{i=1}^n \text {var}(W_{n,i}) ||U_i \left( \hat{\boldsymbol{\beta }}_n, {\alpha } (\hat{\boldsymbol{\beta }}_n, {\phi } (\hat{\boldsymbol{\beta }}_n)) \right) ||^2. \end{aligned}$$

For ε>0 and α>0, when $\frac{r_n^{1/2}}{nm^{1/2}} (\sum _{k=1}^m W_{n,i,k}) U_i \left( \hat{\boldsymbol{\beta }}_n, {\alpha } (\hat{\boldsymbol{\beta }}_n, {\phi } (\hat{\boldsymbol{\beta }}_n)) \right) \ge \varepsilon {Z}_n$,

C20 $$\begin{aligned} \begin{aligned}&({Z}_n^2)^{-1}\sum _{i=1}^n E^* \{ ||\frac{r_n^{1/2}}{nm^{1/2}} (\sum _{k=1}^m W_{n,i,k}) U_i \left( \hat{\boldsymbol{\beta }}_n, {\alpha } (\hat{\boldsymbol{\beta }}_n, {\phi } (\hat{\boldsymbol{\beta }}_n)) \right) ||^2 \\&\times I ( ||\frac{r_n^{1/2}}{nm^{1/2}} (\sum _{k=1}^m W_{n,i,k}) U_i (\hat{\boldsymbol{\beta }}_n) || \ge \varepsilon {Z}_n ) \} \\ \le&(\varepsilon {Z}_n^2)^{-1-\alpha /2} \sum _{i=1}^n \frac{r_n^{1+\alpha /2}}{n^{2+\alpha } m^{1+\alpha /2}} \times E^* \{ ||\sum _{k=1}^m W_{n,i,k} U_i \left( \hat{\boldsymbol{\beta }}_n, {\alpha } (\hat{\boldsymbol{\beta }}_n, {\phi } (\hat{\boldsymbol{\beta }}_n)) \right) ||^{2+\alpha } \\&\times I ( ||\frac{r_n^{1/2}}{nm^{1/2}} (\sum _{k=1}^m W_{n,i,k}) U_i \left( \hat{\boldsymbol{\beta }}_n, {\alpha } (\hat{\boldsymbol{\beta }}_n, {\phi } (\hat{\boldsymbol{\beta }}_n)) \right) || \ge \varepsilon {Z}_n) \} \\ \le&\frac{r_n^{1+\alpha /2}}{n^{2+\alpha } m^{1+\alpha /2} \varepsilon ^{1+\alpha /2} {Z}_n^{2+\alpha }} \sum _{i=1}^n E^* \left( || \sum _{k=1}^m W_{n,i,k} U_i \left( \hat{\boldsymbol{\beta }}_n, {\alpha } (\hat{\boldsymbol{\beta }}_n, {\phi } (\hat{\boldsymbol{\beta }}_n)) \right) ||^{2+\alpha } \right) \\ \le&\frac{r_n^{1+\alpha /2} E(\sum _{k=1}^m \max _{i\le n} W_{n,i,k})^{2+\alpha } }{n^{1+\alpha } m^{1+\alpha /2} \varepsilon ^{1+\alpha /2} {Z}_n^{2+\alpha }} \left( \frac{1}{n}\sum _{i=1}^n || U_i \left( \hat{\boldsymbol{\beta }}_n, {\alpha } (\hat{\boldsymbol{\beta }}_n, {\phi } (\hat{\boldsymbol{\beta }}_n)) \right) ||^{2+\alpha } \right) . \end{aligned} \end{aligned}$$

Note that $\limsup _{n \rightarrow \infty }\frac{ r_n^{1+\alpha /2} E(\sum _{k=1}^{m} \max _{i\le n} W_{n,i,k} )^{2+\alpha }}{n^{1+\alpha }m^{1+\alpha /2} \varepsilon ^{1+\alpha /2}} = 0$ as $r_n, n \rightarrow \infty$ under the Assumption 5. Then the left side of the (C20) goes to zero as $r_n,n \rightarrow \infty$. Hence the Lindeberg condition is satisfied. By the Lindeberg-Feller central limit theorem, the asymptotic distribution of (C19) is a multivariate normal with zero mean and covariance matrix

$$\begin{aligned} \lim _{n \rightarrow \infty } \frac{r_n}{n} \left( \frac{1}{n}\sum _{i=1}^n \text {var} (W_{n,i}) D_i (\hat{\boldsymbol{\beta }}_n)^T V_i (\hat{\boldsymbol{\beta }}_n)^{-1} (Y_i - \mu _i(\hat{\boldsymbol{\beta }}_n) )^2V_i (\hat{\boldsymbol{\beta }}_n)^{-1}D_i (\hat{\boldsymbol{\beta }}_n) \right) \end{aligned}$$

where $$\text {var} (W_{n,i}) = \frac{1}{q_{n,i}}-1+b_{n,i}^2q_{n,i}, i=1,\ldots , n$$. Therefore,

$$\sum _{i=1}^n \frac{r_n^{1/2}}{nm^{1/2}} ( \sum _{k=1}^m W_{n,i,k}) U_i \left( \hat{\boldsymbol{\beta }}_n, \tilde{\alpha }_{r_n} (\hat{\boldsymbol{\beta }}_n, \tilde{\phi }_{r_n} ( \hat{\boldsymbol{\beta }}_n ) ) \right) =O_{p^*}(1)$$.

Applying the Slutsky’s theorem to Equation (C17) , we can obtain the corresponding asymptotic distribution of $(r_n m )^{1/2} (\tilde{\boldsymbol{\beta }}_n^{ (m)} - \hat{\boldsymbol{\beta }}_n)$, which is a multivariate normal with zero mean and covariance matrix

$$\begin{aligned} \lim _{n \rightarrow \infty } r_n&\left( \sum _{i=1}^n (Q_i (\hat{\boldsymbol{\beta }}_n) + D_i (\hat{\boldsymbol{\beta }}_n)^TV_i (\hat{\boldsymbol{\beta }}_n)^{-1}D_i (\hat{\boldsymbol{\beta }}_n) )\right) ^{-1}\\ \times&\left( \sum _{i=1}^n (\frac{1}{q_{n,i}}-1+b_{n,i}^2q_{n,i}) D_i (\hat{\boldsymbol{\beta }}_n)^T V_i (\hat{\boldsymbol{\beta }}_n)^{-1} (Y_i - \mu _i(\hat{\boldsymbol{\beta }}_n) )^2V_i (\hat{\boldsymbol{\beta }}_n)^{-1}D_i (\hat{\boldsymbol{\beta }}_n) \right) \\&\left( \sum _{i=1}^n (Q_i (\hat{\boldsymbol{\beta }}_n) + D_i (\hat{\boldsymbol{\beta }}_n)^TV_i (\hat{\boldsymbol{\beta }}_n)^{-1}D_i (\hat{\boldsymbol{\beta }}_n)) \right) ^{-1} \end{aligned}$$

As a special case, when $q_{n,i} = \frac{r_n}{n}$ and $\text {var}(G_{n,i}) = b_n^2$ for i=1,…,n, then the conditional variance-covariance matrix of $(r_nm)^{1/2} (\tilde{\boldsymbol{\beta }}_n^{(m)} - \hat{\boldsymbol{\beta }}_n)$ can be written as

$$\begin{aligned} \lim _{n \rightarrow \infty } g(r_n,n)&\left( \sum _{i=1}^n (Q_i (\hat{\boldsymbol{\beta }}_n) + D_i (\hat{\boldsymbol{\beta }}_n)^TV_i (\hat{\boldsymbol{\beta }}_n)^{-1}D_i (\hat{\boldsymbol{\beta }}_n) )\right) ^{-1}\\ \times&\left( \sum _{i=1}^n D_i (\hat{\boldsymbol{\beta }}_n)^T V_i (\hat{\boldsymbol{\beta }}_n)^{-1} (Y_i - \mu _i(\hat{\boldsymbol{\beta }}_n) )^2V_i (\hat{\boldsymbol{\beta }}_n)^{-1}D_i (\hat{\boldsymbol{\beta }}_n) \right) \\&\left( \sum _{i=1}^n (Q_i (\hat{\boldsymbol{\beta }}_n) + D_i (\hat{\boldsymbol{\beta }}_n)^TV_i (\hat{\boldsymbol{\beta }}_n)^{-1}D_i (\hat{\boldsymbol{\beta }}_n)) \right) ^{-1} \end{aligned}$$

where factor $g(r_n,n) = n-r_n + b_n^2 r_n^2/n$. This completes the proof.

## Appendix D Proof of Theorem A.3

The distance between $\tilde{\boldsymbol{\beta }}_n$ around ${\boldsymbol{\beta }}_0$ can also be quantified. Unconditionally the randomness come from both the stochastic weights and data. Do Taylor expansion with the subset generalized estimating function around the true value of parameter:

$$\begin{aligned} 0 =&\sum _{i=1}^n W_{n,i} D_i (\tilde{\boldsymbol{\beta }}_n) V_i^{-1} (\tilde{\alpha }_{r_n} (\tilde{\boldsymbol{\beta }}_n, \tilde{\phi }_{r_n} (\tilde{\boldsymbol{\beta }}_n)) (Y_i - \mu _i(\tilde{\boldsymbol{\beta }}_n)) \\ =&\sum _{i=1}^n W_{n,i} D_i ({\boldsymbol{\beta }}_0) V_i^{-1} (\tilde{\alpha }_{r_n} ({\boldsymbol{\beta }}_0, \tilde{\phi }_{r_n} ( {\boldsymbol{\beta }}_0)) (Y_i - \mu _i({\boldsymbol{\beta }}_0) ) \\ +&\sum _{i=1}^n W_{n,i} (\tilde{\boldsymbol{\beta }}_n- {\boldsymbol{\beta }}_0) \left( \tilde{Q}_i (\boldsymbol{\beta }^*) - D_i (\boldsymbol{\beta }^*) V_i^{-1} (\tilde{\alpha }_{r_n} (\boldsymbol{\beta }^*, \tilde{\phi }_{r_n} ( \boldsymbol{\beta }^*))D_i (\boldsymbol{\beta }^*) \right) \end{aligned}$$

where $\tilde{Q}_i$ is a p×p matrix given by

$$\begin{aligned} \tilde{Q}_i (\boldsymbol{\beta }) = \frac{\partial D_i (\boldsymbol{\beta }) V_i^{-1} (\tilde{\alpha }_{r_n} (\boldsymbol{\beta }, \tilde{\phi }_{r_n} (\boldsymbol{\beta })) }{\partial \boldsymbol{\beta }^T} (Y_i-\mu _i({\boldsymbol{\beta }})) \end{aligned}$$

and $\boldsymbol{\beta }^*$ is some value between $\tilde{\boldsymbol{\beta }}_n$ and ${\boldsymbol{\beta }}_0$. Then we obtain:

D21 $$\begin{aligned} \begin{aligned} r_n^{1/2} (\tilde{\boldsymbol{\beta }}_n - {\boldsymbol{\beta }}_0) =&\left( \frac{1}{n} \sum _{i=1}^n W_{n,i} \left( -\tilde{Q}_i (\boldsymbol{\beta }^*) + D_i (\boldsymbol{\beta }^*) V_i^{-1} (\tilde{\alpha }_{r_n} (\boldsymbol{\beta }^*, \tilde{\phi }_{r_n} ( \boldsymbol{\beta }^*))D_i (\boldsymbol{\beta }^*) \right) \right) ^{-1}\\&\times \frac{r_n^{1/2}}{n} \left( \sum _{i=1}^n W_{n,i} U_i \left( {\boldsymbol{\beta }}_0, \tilde{\alpha }_{r_n} ({\boldsymbol{\beta }}_0, \tilde{\phi }_{r_n} ( {\boldsymbol{\beta }}_0 )) \right) \right) \end{aligned} \end{aligned}$$

where $U_i \left( {\boldsymbol{\beta }}_0, \tilde{\alpha }_{r_n} ({\boldsymbol{\beta }}_0, \tilde{\phi }_{r_n} ( {\boldsymbol{\beta }}_0 ) ) \right) = D_i ({\boldsymbol{\beta }}_0) V_i^{-1} (\tilde{\alpha }_{r_n} ({\boldsymbol{\beta }}_0, \tilde{\phi }_{r_n} ( {\boldsymbol{\beta }}_0)) (Y_i - \mu _i({\boldsymbol{\beta }}_0) )$ By continuous mapping, as $r_n, n \rightarrow \infty$,

D22 $$\begin{aligned} \begin{aligned}&\frac{1}{n} \sum _{i=1}^n W_{n,i} \left( -\tilde{Q}_i (\boldsymbol{\beta }^*) + D_i (\boldsymbol{\beta }^*) V_i^{-1} (\tilde{\alpha }_{r_n} (\boldsymbol{\beta }^*, \tilde{\phi }_{r_n} ( \boldsymbol{\beta }^*))D_i (\boldsymbol{\beta }^*) \right) \\ =&\frac{1}{n} \sum _{i=1}^n W_{n,i} \left( -\tilde{Q}_i ({\boldsymbol{\beta }}_0) + D_i ({\boldsymbol{\beta }}_0) V_i^{-1} (\tilde{\alpha }_{r_n} ({\boldsymbol{\beta }}_0, \tilde{\phi }_{r_n} ( {\boldsymbol{\beta }}_0))D_i ({\boldsymbol{\beta }}_0) \right) + o_p(1). \end{aligned} \end{aligned}$$

Next we investigate the consistency of the nuisance parameter, note that

D23 $$\begin{aligned} V_i^{-1} (\tilde{\alpha }_{r_n} (\boldsymbol{\beta }_0, \tilde{\phi }_{r_n} ( \boldsymbol{\beta }_0)) = V_i^{-1} ({\alpha } (\boldsymbol{\beta }_0, {\phi } ( \boldsymbol{\beta }_0)) + r_n^{-1/2} \frac{\partial }{\partial \alpha } \left( V_i^{-1} ({\alpha } (\boldsymbol{\beta }_0, {\phi } ( \boldsymbol{\beta }_0)) \right) r_n^{1/2} (\alpha ^*-\alpha ), \end{aligned}$$

where $\alpha ^*$ is some value between $\tilde{\alpha }_{r_n}$ and α and

$$\begin{aligned} r_n^{1/2} (\alpha ^*-\alpha ) =&r_n^{1/2} (\alpha ^* (\boldsymbol{\beta }_0, {\phi }^* ( \boldsymbol{\beta }_0))-\alpha )\\ =&r_n^{1/2} (\alpha ^* (\boldsymbol{\beta }_0, {\phi }^* (\boldsymbol{\beta }_0))-\alpha ^* ( \boldsymbol{\beta }_0, {\phi } ( \boldsymbol{\beta }_0)) + \alpha ^* ( \boldsymbol{\beta }_0, {\phi } ( \boldsymbol{\beta }_0)) - \alpha )\\ =&r_n^{1/2}\left( \frac{\partial \alpha ^*}{\partial \phi } (\boldsymbol{\beta }_0, {\phi }^{**}) ({\phi }^*-\phi ) \right) + r_n^{1/2}\left( \alpha ^* ( \boldsymbol{\beta }_0, {\phi } (\boldsymbol{\beta }_0)) - \alpha \right) = O_{p} (1) \end{aligned}$$

by the consistency of $\tilde{\alpha }_{r_n}$ and $\tilde{\phi }_{r_n}$ under Assumption 3. Hence under Assumption 5, we have

$V_i^{-1} (\tilde{\alpha }_{r_n} (\hat{\boldsymbol{\beta }}_n, \tilde{\phi }_{r_n} ( \hat{\boldsymbol{\beta }}_n)) = V_i^{-1} ({\alpha } (\hat{\boldsymbol{\beta }}_n, {\phi } ( \hat{\boldsymbol{\beta }}_n)) + O_{p} (r_n^{-1/2})$ and therefore as $r_n, n \rightarrow \infty$,

D24 $$\begin{aligned} \begin{aligned}&\frac{1}{n} \sum _{i=1}^n W_{n,i} \left( -\tilde{Q}_i ({\boldsymbol{\beta }}_0) + D_i ({\boldsymbol{\beta }}_0) V_i^{-1} (\tilde{\alpha }_{r_n} ({\boldsymbol{\beta }}_0, \tilde{\phi }_{r_n} ( {\boldsymbol{\beta }}_0))D_i ({\boldsymbol{\beta }}_0) \right) \\ =&\frac{1}{n} \sum _{i=1}^n W_{n,i} \left( -{Q}_i ({\boldsymbol{\beta }}_0) + D_i ({\boldsymbol{\beta }}_0) V_i^{-1} ({\alpha } ({\boldsymbol{\beta }}_0, {\phi } ( {\boldsymbol{\beta }}_0))D_i ({\boldsymbol{\beta }}_0) \right) + o_p(1). \end{aligned} \end{aligned}$$

where $Q_i$ is a p×p matrix given by

$$\begin{aligned} Q_i (\boldsymbol{\beta }) = \frac{\partial D_i (\boldsymbol{\beta }) V_i^{-1} (\boldsymbol{\beta })}{\partial \boldsymbol{\beta }^T} (Y_i-\mu _i({\boldsymbol{\beta }}) ). \end{aligned}$$

By the strong law of large numbers, as $r_n, n \rightarrow \infty$,

D25 $$\begin{aligned} \begin{aligned}&\frac{1}{n} \sum _{i=1}^n W_{n,i} \left( -{Q}_i ({\boldsymbol{\beta }}_0) + D_i ({\boldsymbol{\beta }}_0) V_i^{-1} ({\alpha } ({\boldsymbol{\beta }}_0, {\phi } ( {\boldsymbol{\beta }}_0))D_i ({\boldsymbol{\beta }}_0) \right) = \\&E D (\boldsymbol{\beta }_0) V^{-1} ({\alpha } (\boldsymbol{\beta }_0, {\phi } ( \boldsymbol{\beta }_0))D (\boldsymbol{\beta }_0) + o_p(1). \end{aligned} \end{aligned}$$

Combine equation (D22), (D24) and (D25), we have

D26 $$\begin{aligned} \begin{aligned}&\frac{1}{n} \sum _{i=1}^n W_{n,i} \left( -\tilde{Q}_i (\boldsymbol{\beta }^*) + D_i (\boldsymbol{\beta }^*) V_i^{-1} (\tilde{\alpha }_{r_n} (\boldsymbol{\beta }^*, \tilde{\phi }_{r_n} ( \boldsymbol{\beta }^*))D_i (\boldsymbol{\beta }^*) \right) = \\&E D (\boldsymbol{\beta }_0) V^{-1} ({\alpha } (\boldsymbol{\beta }_0, {\phi } ( \boldsymbol{\beta }_0))D (\boldsymbol{\beta }_0) + o_p(1). \end{aligned} \end{aligned}$$

From Theorem ??, the proposed estimator $\tilde{\boldsymbol{\beta }}_n^{ (m)}$ is ${(r_n m )^{1/2}}$ consistent to the full data estimator condition on the data. By the triangle inequality,

$$\begin{aligned} ||\tilde{\boldsymbol{\beta }}_n^{ (m)} - {\boldsymbol{\beta }}_0|| \le ||\tilde{\boldsymbol{\beta }}_n^{ (m)} - \hat{\boldsymbol{\beta }}_n|| + ||\hat{\boldsymbol{\beta }}_n - {\boldsymbol{\beta }}_0||. \end{aligned}$$

When $r_n m < n$, the term $||\tilde{\boldsymbol{\beta }}_n^{ (m)} - \hat{\boldsymbol{\beta }}_n||$ which is in the order of $O_p ((r_nm)^{-1/2})$ dominates, hence $\tilde{\boldsymbol{\beta }}_n^{ (m)}$ is root-$(r_nm)$ consistent to the true value. While $r_n m \ge n$, the term $||\hat{\boldsymbol{\beta }}_n - {\boldsymbol{\beta }}_0||$ which is in the order of $O_p (n^{-1/2})$ dominates, hence $\tilde{\boldsymbol{\beta }}_n^{ (m)}$ is root-*n* consistent to the true value. We will analyze them seperately.

(1) When $r_n m < n$, given that $\tilde{\boldsymbol{\beta }}_n^{ (m)} = \frac{1}{m} \sum _{k=1}^m \tilde{\boldsymbol{\beta }}_{n,k}$ and equation (D21), (D26)

D27 $$\begin{aligned} \begin{aligned} (r_n m )^{1/2} (\tilde{\boldsymbol{\beta }}_n^{ (m)} - {\boldsymbol{\beta }}_0)&= m^{-1/2} \sum _{k=1}^m \left( r_n^{1/2} (\tilde{\boldsymbol{\beta }}_{n,k} - {\boldsymbol{\beta }}_0) \right) \\&= \left( E D (\boldsymbol{\beta }_0) V^{-1} ({\alpha } (\boldsymbol{\beta }_0, {\phi } ( \boldsymbol{\beta }_0))D (\boldsymbol{\beta }_0) \right) ^{-1}\\&\quad \times \sum _{i=1}^n \frac{r_n^{1/2}}{nm^{1/2}} \left( \sum _{k=1}^m W_{n,i,k} U_i \left( \boldsymbol{\beta }_0, \tilde{\alpha }_{r_n} (\boldsymbol{\beta }_0, \tilde{\phi }_{r_n} ( \boldsymbol{\beta }_0 ) ) \right) \right) \\&\quad + o_{p}\left( \sum _{i=1}^n \frac{r_n^{1/2}}{nm^{1/2}} \left( \sum _{k=1}^m W_{n,i,k} U_i \left( \boldsymbol{\beta }_0, \tilde{\alpha }_{r_n} (\boldsymbol{\beta }_0, \tilde{\phi }_{r_n} ( \boldsymbol{\beta }_0 ) ) \right) \right) \right) . \end{aligned} \end{aligned}$$

Now we show that $\sum _{i=1}^n \frac{r_n^{1/2}}{nm^{1/2}} ( \sum _{k=1}^m W_{n,i,k}) U_i \left( \boldsymbol{\beta }_0, \tilde{\alpha }_{r_n} (\boldsymbol{\beta }_0, \tilde{\phi }_{r_n} ( \boldsymbol{\beta }_0 ) ) \right) =O_{p}(1)$. Given (D23), we have

D28 $$\begin{aligned} \begin{aligned}&\sum _{i=1}^n \frac{r_n^{1/2}}{nm^{1/2}} \left( \sum _{k=1}^m W_{n,i,k} U_i \left( \boldsymbol{\beta }_0, \tilde{\alpha }_{r_n} (\boldsymbol{\beta }_0, \tilde{\phi }_{r_n} ( \boldsymbol{\beta }_0 ) ) \right) \right) \\&\quad = \frac{r_n^{1/2}}{nm^{1/2}} \left( \sum _{i=1}^n (\sum _{k=1}^m W_{n,i,k}) U_i \left( \boldsymbol{\beta }_0, {\alpha } ({\boldsymbol{\beta }}_0, {\phi } ({\boldsymbol{\beta }}_0)) \right) \right) \\&\qquad + r_n^{1/2} (\alpha ^*-\alpha ) \frac{1}{nm^{1/2}}\sum _{i=1}^n (\sum _{k=1}^m W_{n,i,k}) \frac{\partial }{\partial \alpha } U_i \left( \boldsymbol{\beta }_0, {\alpha } ({\boldsymbol{\beta }}_0, {\phi } ({\boldsymbol{\beta }}_0)) \right) \end{aligned} \end{aligned}$$

where $U_i \left( \boldsymbol{\beta }_0, {\alpha } ({\boldsymbol{\beta }}_0, {\phi } ({\boldsymbol{\beta }}_0)) \right) = D_i ({\boldsymbol{\beta }}_0) V_i^{-1} ({\alpha } ({\boldsymbol{\beta }}_0, {\phi } ( {\boldsymbol{\beta }}_0)) (Y_i - \mu _i({\boldsymbol{\beta }}_0))$. Notice that

$$\begin{aligned} E\left( \frac{1}{nm^{1/2}}\sum _{i=1}^n (\sum _{k=1}^m W_{n,i,k}) \frac{\partial }{\partial \alpha } U_i \left( \boldsymbol{\beta }_0, {\alpha } ({\boldsymbol{\beta }}_0, {\phi } ({\boldsymbol{\beta }}_0)) \right) \right) =&\frac{\partial }{\partial \alpha } \left( \frac{m^{1/2}}{n}\sum _{i=1}^n U_i \left( \boldsymbol{\beta }_0, {\alpha } ({\boldsymbol{\beta }}_0, {\phi } ({\boldsymbol{\beta }}_0)) \right) \right) = 0 \end{aligned}$$

by Theorem 7.17 of [48], 3rd Edition, under Assumption 3. Also,

$$\begin{aligned} \text {var} \left( \frac{1}{nm^{1/2}}\sum _{i=1}^n (\sum _{k=1}^m W_{n,i,k}) \frac{\partial U_i }{\partial \alpha } \right) = \left( \frac{1}{n^2} \sum _{i=1}^n (\text {var} (W_{n,i}) + m) \right) E(\frac{\partial U_i }{\partial \alpha })^2 \end{aligned}$$

because $E(W_{n,i})=1$, $E(\frac{\partial U_i }{\partial \alpha })^2 = \text {var}(\frac{\partial U_i }{\partial \alpha })$ due to interchange of integral and differentiation, which is established by the Dominated Convergence Theorem under Assumption 3. When $r_nm < n$ and $\lim \sup _{n\rightarrow \infty }\text {var}(W_{n,i})/\frac{n}{r_n} < \infty$, it is easy to see that

$$\limsup _{n\rightarrow \infty }\text {var} \left( \frac{1}{nm^{1/2}}\sum _{i=1}^n (\sum _{k=1}^m W_{n,i,k}) \frac{\partial U_i }{\partial \alpha } \right) /\frac{1}{r_n}<\infty $$. Hence in (D28),

$$r_n^{1/2} (\alpha ^*-\alpha ) \frac{1}{nm^{1/2}}\sum _{i=1}^n (\sum _{k=1}^m W_{n,i,k}) \frac{\partial }{\partial \alpha } U_i \left( \boldsymbol{\beta }_0, {\alpha } ({\boldsymbol{\beta }}_0, {\phi } ({\boldsymbol{\beta }}_0)) \right) = o_{p}(1) $$. The term

$\sum _{i=1}^n \frac{r_n^{1/2}}{nm^{1/2}} \left( \sum _{k=1}^m W_{n,i,k} U_i \left( \boldsymbol{\beta }_0, \tilde{\alpha }_{r_n} (\boldsymbol{\beta }_0, \tilde{\phi }_{r_n} ( \boldsymbol{\beta }_0 ) ) \right) \right)$ is asymptotically equivalent to

D29 $$\begin{aligned} \sum _{i=1}^n \frac{r_n^{1/2}}{nm^{1/2}} (\sum _{k=1}^m W_{n,i,k}) U_i \left( \boldsymbol{\beta }_0, {\alpha } (\boldsymbol{\beta }_0, {\phi } (\boldsymbol{\beta }_0)) \right) , \end{aligned}$$

which is a sum of *n* independent elements, with *i*th element equal to

$$\frac{r_n^{1/2}}{nm^{1/2}} (\sum _{k=1}^m W_{n,i,k}) U_i \left( \boldsymbol{\beta }_0, {\alpha } (\boldsymbol{\beta }_0, {\phi } (\boldsymbol{\beta }_0)) \right) $$.

Define

$$\begin{aligned} {S}_n^2&= \left| \left| \sum _{i=1}^n \text {var} \left( \frac{r_n^{1/2}}{nm^{1/2}} (\sum _{k=1}^m W_{n,i,k}) U_i ({\boldsymbol{\beta }}_0 ) \right) \right| \right| \\&=\frac{r_n}{n^2}\sum _{i=1}^n (\text {var} (W_{n,i}) + m) E||U_i \left( {\boldsymbol{\beta }}_0, {\alpha } ({\boldsymbol{\beta }}_0, {\phi } ({\boldsymbol{\beta }}_0)) \right) ||^2. \end{aligned}$$

For ε>0 and α>0, when $\frac{r_n^{1/2}}{nm^{1/2}} (\sum _{k=1}^m W_{n,i,k}) U_i \left( {\boldsymbol{\beta }}_0, {\alpha } ({\boldsymbol{\beta }}_0, {\phi } ({\boldsymbol{\beta }}_0)) \right) \ge \varepsilon {S}_n$,

D30 $$\begin{aligned} \begin{aligned}&({S}_n^2)^{-1}\sum _{i=1}^n E \{ ||\frac{r_n^{1/2}}{nm^{1/2}} (\sum _{k=1}^m W_{n,i,k}) U_i \left( {\boldsymbol{\beta }}_0, {\alpha } ({\boldsymbol{\beta }}_0, {\phi } ({\boldsymbol{\beta }}_0)) \right) ||^2 \\&\times I ( ||\frac{r_n^{1/2}}{nm^{1/2}} (\sum _{k=1}^m W_{n,i,k}) U_i ({\boldsymbol{\beta }}_0)|| \ge \varepsilon {S}_n ) \}\\ \le&(\varepsilon {S}_n^2)^{-1-\alpha /2} \sum _{i=1}^n \frac{r_n^{1+\alpha /2}}{n^{2+\alpha } m^{1+\alpha /2}} E \{||\sum _{k=1}^m W_{n,i,k} U_i \left( {\boldsymbol{\beta }}_0, {\alpha } ({\boldsymbol{\beta }}_0, {\phi } ({\boldsymbol{\beta }}_0)) \right) ||^{2+\alpha } \\&\times I ( ||\frac{r_n^{1/2}}{nm^{1/2}} (\sum _{k=1}^m W_{n,i,k}) U_i \left( {\boldsymbol{\beta }}_0, {\alpha } ({\boldsymbol{\beta }}_0, {\phi } ({\boldsymbol{\beta }}_0)) \right) || \ge \varepsilon {S}_n) \} \\ \le&\frac{r_n^{1+\alpha /2}}{n^{2+\alpha } m^{1+\alpha /2} \varepsilon ^{1+\alpha /2} {S}_n^{2+\alpha }} \sum _{i=1}^n E \left( || \sum _{k=1}^m W_{n,i,k} U_i \left( {\boldsymbol{\beta }}_0, {\alpha } ({\boldsymbol{\beta }}_0, {\phi } ({\boldsymbol{\beta }}_0)) \right) ||^{2+\alpha } \right) \\ \le&\frac{ r_n^{1+\alpha /2} E(\sum _{k=1}^m \max _{i\le n} W_{n,i,k} )^{2+\alpha } }{n^{2+\alpha } m^{1+\alpha /2} \varepsilon ^{1+\alpha /2} {S}_n^{2+\alpha } } \left( \frac{1}{n}\sum _{i=1}^n || U_i \left( {\boldsymbol{\beta }}_0, {\alpha } ({\boldsymbol{\beta }}_0, {\phi } ({\boldsymbol{\beta }}_0)) \right) ||^{2+\alpha } \right) . \end{aligned} \end{aligned}$$

Note that $\limsup _{n \rightarrow \infty }\frac{ r_n^{1+\alpha /2} E(\sum _{k=1}^m \max _{i\le n} W_{n,i,k} )^{2+\alpha } }{n^{2+\alpha } m^{1+\alpha /2} \varepsilon ^{1+\alpha /2}} = 0$ when $r_nm < n$ as $r_n, n \rightarrow \infty$ under the Assumption 5. Then (D30) goes to zero as $r_n,n \rightarrow \infty$. Hence the Lindeberg condition is satisfied. By the Lindeberg-Feller central limit theorem, the asymptotic distribution of (D29) is a multivariate normal with zero mean and covariance matrix

$$\begin{aligned} \lim _{n \rightarrow \infty } \frac{r_n}{n} \left( \frac{1}{n}\sum _{i=1}^n (\text {var} (W_{n,i}) + m) D_i^T V_i^{-1} \text {cov}(Y_i) V_i^{-1}D_i \right) \end{aligned}$$

where $$\text {var} (W_{n,i}) = \frac{1}{q_{n,i}}-1+b_{n,i}^2q_{n,i}, i=1,\ldots , n$$. Therefore,

$$\sum _{i=1}^n \frac{r_n^{1/2}}{nm^{1/2}} ( \sum _{k=1}^m W_{n,i,k}) U_i \left( {\boldsymbol{\beta }}_0, \tilde{\alpha }_{r_n} ({\boldsymbol{\beta }}_0, \tilde{\phi }_{r_n} ( {\boldsymbol{\beta }}_0 ) ) \right) =O_{p}(1)$$.

Applying the Slutsky’s theorem to Equation (D27), we can obtain the corresponding asymptotic distribution of $(r_n m )^{1/2} (\tilde{\boldsymbol{\beta }}_n^{ (m)} - {\boldsymbol{\beta }}_0)$, which is a multivariate normal with zero mean and covariance matrix

$$\begin{aligned}&\lim _{n \rightarrow \infty } \frac{r_n}{n} (\sum _{i=1}^n D_i^TV_i^{-1}D_i)^{-1} \left( \sum _{i=1}^n (\frac{1}{q_{n,i}}-1+b_{n,i}^2q_{n,i} + m) D_i^T V_i^{-1} \text {cov}(Y_i) V_i^{-1}D_i \right) \\&\quad \times (\sum _{i=1}^n D_i^TV_i^{-1}D_i)^{-1}. \end{aligned}$$

(2) When $r_nm>n$, given that $\tilde{\boldsymbol{\beta }}_n^{ (m)} = \frac{1}{m} \sum _{k=1}^m \tilde{\boldsymbol{\beta }}_{n,k}$ and equation (D21), (D26), we have

D31 $$\begin{aligned} \begin{aligned} n^{1/2} (\tilde{\boldsymbol{\beta }}_n^{ (m)} - {\boldsymbol{\beta }}_0)&= \frac{n^{1/2}}{r_n^{1/2}m } \sum _{k=1}^m \left( r_n^{1/2} (\tilde{\boldsymbol{\beta }}_{n,k} - {\boldsymbol{\beta }}_0) \right) \\&= \left( E D (\boldsymbol{\beta }_0) V^{-1} ({\alpha } (\boldsymbol{\beta }_0, {\phi } ( \boldsymbol{\beta }_0))D (\boldsymbol{\beta }_0) \right) ^{-1} \\&\quad \times \sum _{i=1}^n \frac{1}{n^{1/2}m} \left( \sum _{k=1}^m W_{n,i,k} U_i \left( \boldsymbol{\beta }_0, \tilde{\alpha }_{r_n} (\boldsymbol{\beta }_0, \tilde{\phi }_{r_n} ( \boldsymbol{\beta }_0 ) ) \right) \right) \\&\qquad + o_{p}\left( \sum _{i=1}^n \frac{1}{n^{1/2}m} \left( \sum _{k=1}^m W_{n,i,k} U_i \left( \boldsymbol{\beta }_0, \tilde{\alpha }_{r_n} (\boldsymbol{\beta }_0, \tilde{\phi }_{r_n} ( \boldsymbol{\beta }_0 ) ) \right) \right) \right) . \end{aligned} \end{aligned}$$

where $W_{n,i,k}$ represents the random weight assigned to the *i*th subject in *k*th perturbation. Now we show that $\sum _{i=1}^n \frac{1}{n^{1/2}m} \left( \sum _{k=1}^m W_{n,i,k} U_i \left( \boldsymbol{\beta }_0, \tilde{\alpha }_{r_n} (\boldsymbol{\beta }_0, \tilde{\phi }_{r_n} ( \boldsymbol{\beta }_0 ) ) \right) \right) =O_{p}(1)$. Notice that

$$\begin{aligned} \text {var} \left( \frac{1}{n^{1/2}m r_n^{1/2}}\sum _{i=1}^n (\sum _{k=1}^m W_{n,i,k}) \frac{\partial U_i }{\partial \alpha } \right) = \left( \frac{1}{nmr_n} \sum _{i=1}^n (\text {var} (W_{n,i}) + m) \right) E(\frac{\partial U_i }{\partial \alpha })^2. \end{aligned}$$

When $$r_nm > n$$ and $$\lim \sup _{n\rightarrow \infty }\text {var}(W_{n,i})/\frac{n}{r_n} < \infty $$,

$\limsup _{n\rightarrow \infty }\text {var} \left( \frac{1}{n^{1/2}mr_n^{1/2}}\sum _{i=1}^n (\sum _{k=1}^m W_{n,i,k}) \frac{\partial U_i }{\partial \alpha } \right) /\frac{1}{r_n}<\infty$. Hence similar to (D28),

$$r_n^{1/2} (\alpha ^*-\alpha ) \frac{1}{nm^{1/2}r_n^{1/2}}\sum _{i=1}^n (\sum _{k=1}^m W_{n,i,k}) \frac{\partial }{\partial \alpha } U_i \left( \boldsymbol{\beta }_0, {\alpha } ({\boldsymbol{\beta }}_0, {\phi } ({\boldsymbol{\beta }}_0)) \right) = o_{p}(1) $$. The term

$\sum _{i=1}^n \frac{1}{n^{1/2}m } \left( \sum _{k=1}^m W_{n,i,k} U_i \left( \boldsymbol{\beta }_0, \tilde{\alpha }_{r_n} (\boldsymbol{\beta }_0, \tilde{\phi }_{r_n} ( \boldsymbol{\beta }_0 ) ) \right) \right)$ is asymptotically equivalent to

D32 $$\begin{aligned} \sum _{i=1}^n \frac{1}{n^{1/2}m } (\sum _{k=1}^m W_{n,i,k}) U_i \left( \boldsymbol{\beta }_0, {\alpha } (\boldsymbol{\beta }_0, {\phi } (\boldsymbol{\beta }_0)) \right) , \end{aligned}$$

which is a sum of *n* independent elements, with *i*th element equal to

$$ \frac{1}{n^{1/2}m } (\sum _{k=1}^m W_{n,i,k}) U_i \left( \boldsymbol{\beta }_0, {\alpha } (\boldsymbol{\beta }_0, {\phi } (\boldsymbol{\beta }_0)) \right) $$.

Define

$$\begin{aligned} {T}_n^2 =&\left| \left| \sum _{i=1}^n \text {var} \left( \frac{1}{n^{1/2}m } (\sum _{k=1}^m W_{n,i,k}) U_i \left( {\boldsymbol{\beta }}_0, {\alpha } ({\boldsymbol{\beta }}_0, {\phi } ({\boldsymbol{\beta }}_0)) \right) \right) \right| \right| \\ =&\frac{1}{nm}\sum _{i=1}^n (\text {var} (W_{n,i}) + m) E||U_i \left( {\boldsymbol{\beta }}_0, {\alpha } ({\boldsymbol{\beta }}_0, {\phi } ({\boldsymbol{\beta }}_0)) \right) ||^2. \end{aligned}$$

For ε>0 and α>0, when $\frac{1}{n^{1/2}m } (\sum _{k=1}^m W_{n,i,k}) U_i \left( {\boldsymbol{\beta }}_0, {\alpha } ({\boldsymbol{\beta }}_0, {\phi } ({\boldsymbol{\beta }}_0)) \right) \ge \varepsilon {T}_n$,

D33 $$\begin{aligned}  &   ({T}_n^2)^{-1}\sum _{i=1}^n E \left( ||\frac{1}{n^{1/2}m } (\sum _{k=1}^m W_{n,i,k}) U_i \left( {\boldsymbol{\beta }}_0, {\alpha } ({\boldsymbol{\beta }}_0, {\phi } ({\boldsymbol{\beta }}_0)) \right) ||^2 I ( ||\frac{1}{n^{1/2}m } (\sum _{k=1}^m W_{n,i,k}) U_i ({\boldsymbol{\beta }}_0)|| \ge \varepsilon {T}_n ) \right) \nonumber \\  &   \quad \le (\varepsilon {T}_n^2)^{-1-\alpha /2} \sum _{i=1}^n \frac{1}{n^{1+\alpha /2} m^{2+\alpha }} \times \nonumber \\  &   E \left( ||\sum _{k=1}^m W_{n,i,k} U_i \left( {\boldsymbol{\beta }}_0, {\alpha } ({\boldsymbol{\beta }}_0, {\phi } ({\boldsymbol{\beta }}_0)) \right) ||^{2+\alpha } I ( ||\frac{1}{n^{1/2}m } (\sum _{k=1}^m W_{n,i,k}) U_i \left( {\boldsymbol{\beta }}_0, {\alpha } ({\boldsymbol{\beta }}_0, {\phi } ({\boldsymbol{\beta }}_0)) \right) || \ge \varepsilon {T}_n) \right) \nonumber \\  &   \quad \le \frac{1}{n^{1+\alpha /2} m^{2+\alpha } \varepsilon ^{1+\alpha /2} {T}_n^{2+\alpha }} \sum _{i=1}^n E \left( || \sum _{k=1}^m W_{n,i,k} U_i \left( {\boldsymbol{\beta }}_0, {\alpha } ({\boldsymbol{\beta }}_0, {\phi } ({\boldsymbol{\beta }}_0)) \right) ||^{2+\alpha } \right) \nonumber \\  &   \quad \le \frac{ E(\sum _{k=1}^m \max _{i\le n} W_{n,i,k})^{2+\alpha } }{n^{\alpha /2} m^{2+\alpha } \varepsilon ^{1+\alpha /2} {T}_n^{2+\alpha } } \left( \frac{1}{n}\sum _{i=1}^n || U_i \left( {\boldsymbol{\beta }}_0, {\alpha } ({\boldsymbol{\beta }}_0, {\phi } ({\boldsymbol{\beta }}_0)) \right) ||^{2+\alpha } \right) . \end{aligned}$$

Note that $\limsup _{n \rightarrow \infty }\frac{ E(\sum _{k=1}^m \max _{i\le n} W_{n,i,k})^{2+\alpha } }{n^{\alpha /2} m^{2+\alpha } \varepsilon ^{1+\alpha /2} } = 0$ when $r_n m > n$ as $r_n, n \rightarrow \infty$ under the Assumption 5. Then (D33) goes to zero as $r_n,n \rightarrow \infty$. Hence the Lindeberg condition is satisfied. By the Lindeberg-Feller central limit theorem, the asymptotic distribution of (D32) is a multivariate normal with zero mean and covariance matrix

$$\begin{aligned} \lim _{n \rightarrow \infty } \left( \frac{1}{n}\sum _{i=1}^n (\frac{\text {var} (W_{n,i})}{m} + 1) D_i^T V_i^{-1} \text {cov}(Y_i) V_i^{-1}D_i \right) \end{aligned}$$

where $$\text {var} (W_{n,i}) = \frac{1}{q_{n,i}}-1+b_{n,i}^2q_{n,i}, i=1,\ldots , n$$. Therefore,

$$\sum _{i=1}^n \frac{1}{n^{1/2}m } ( \sum _{k=1}^m W_{n,i,k}) U_i \left( {\boldsymbol{\beta }}_0, \tilde{\alpha }_{r_n} ({\boldsymbol{\beta }}_0, \tilde{\phi }_{r_n} ( {\boldsymbol{\beta }}_0 ) ) \right) =O_{p}(1)$$.

Applying the Slutsky’s theorem to Equation (D31), we can obtain the corresponding asymptotic distribution of $n^{1/2} (\tilde{\boldsymbol{\beta }}_n^{ (m)} - {\boldsymbol{\beta }}_0)$, which is a multivariate normal with zero mean and covariance matrix

$$\begin{aligned}&\lim _{n \rightarrow \infty } n (\sum _{i=1}^n D_i^TV_i^{-1}D_i)^{-1}\left( \sum _{i=1}^n (\frac{\frac{1}{q_{n,i}}-1+b_{n,i}^2q_{n,i}}{m} + 1) D_i^T V_i^{-1} \text {cov}(Y_i) V_i^{-1}D_i \right) \\&\quad \times (\sum _{i=1}^n D_i^TV_i^{-1}D_i)^{-1}. \end{aligned}$$

In particular, for Bernoulli sampling with $q_{n,i} = \frac{r_n}{n}$ and $\text {var}(G_{n,i}) = b_n^2$ for i=1,…,n, when $r_n m<n$, the unconditional variance-covariance matrix of $(r_nm)^{1/2} (\tilde{\boldsymbol{\beta }}_n^{ (m)} - \boldsymbol{\beta }_0)$ can be written as

$$\begin{aligned} \lim _{n \rightarrow \infty } s(m, r_n,n) (\sum _{i=1}^n D_i^TV_i^{-1}D_i)^{-1} \left( \sum _{i=1}^n D_i^T V_i^{-1} \text {cov} (Y_i) V_i^{-1}D_i \right) (\sum _{i=1}^n D_i^TV_i^{-1}D_i)^{-1} \end{aligned}$$

where factor $s(m, r_n,n) = n-r_n + b_n^2 r_n^2/n + mr_n$. When $r_nm>n$, the unconditional variance-covariance matrix of $n^{1/2} (\tilde{\boldsymbol{\beta }}_n^{ (m)} - \boldsymbol{\beta }_0)$ can be written as

$$\begin{aligned} \lim _{n \rightarrow \infty } t(m, r_n,n) (\sum _{i=1}^n D_i^TV_i^{-1}D_i)^{-1} \left( \sum _{i=1}^n D_i^T V_i^{-1} \text {cov} (Y_i) V_i^{-1}D_i \right) (\sum _{i=1}^n D_i^TV_i^{-1}D_i)^{-1} \end{aligned}$$

where $t(m, r_n,n) = n+\frac{n^2}{m r_n} (1-r_n/n+b_n^2r_n^2/n^2)$. This completes the proof.

## Appendix E Simulation and real data analysis result

**Table 5:** Empirical coverage probabilities of $\hat{\beta }_{1}$ based on proposed conditional variance estimation method with different subset sizes for generalized linear regression model with identity link with α=0.3 for $\hbox {m}^*$=50, $\hbox {m}^\dagger$=200, $\hbox {m}^\wr$ = 500 based on N=1,000 simulations

X/Corr	Size *r*	$\hbox {geom}^*$	$\hbox {beta}^*$	$\hbox {gamma}^*$	$\hbox {geom}^\dagger$	$\hbox {beta}^\dagger$	$\hbox {gamma}^\dagger$	$\hbox {geom}^\wr$	$\hbox {beta}^\wr$	$\hbox {gamma}^\wr$
NORM/CS	200	0.946	0.955	0.938	0.950	0.952	0.939	0.944	0.949	0.946
	500	0.944	0.944	0.950	0.952	0.940	0.965	0.960	0.958	0.953
	800	0.929	0.948	0.943	0.949	0.941	0.939	0.954	0.946	0.926
	1000	0.951	0.932	0.938	0.949	0.949	0.948	0.947	0.945	0.943
	1200	0.951	0.950	0.948	0.963	0.951	0.961	0.965	0.955	0.953
	1500	0.935	0.948	0.946	0.951	0.936	0.954	0.957	0.942	0.949
	1800	0.935	0.946	0.940	0.947	0.943	0.949	0.935	0.950	0.940
	2000	0.944	0.951	0.945	0.949	0.947	0.938	0.948	0.944	0.950
	3000	0.949	0.944	0.949	0.948	0.951	0.960	0.938	0.943	0.939
	5000	0.931	0.943	0.957	0.946	0.965	0.949	0.957	0.953	0.959
T/CS	200	0.924	0.935	0.927	0.925	0.914	0.909	0.883	0.861	0.861
	500	0.928	0.940	0.941	0.918	0.917	0.925	0.882	0.859	0.849
	800	0.937	0.941	0.926	0.920	0.924	0.918	0.870	0.871	0.858
	1000	0.939	0.932	0.940	0.906	0.929	0.922	0.887	0.862	0.899
	1200	0.940	0.925	0.925	0.910	0.912	0.922	0.879	0.898	0.891
	1500	0.934	0.943	0.934	0.930	0.933	0.954	0.902	0.902	0.885
	1800	0.934	0.935	0.946	0.935	0.939	0.936	0.905	0.886	0.913
	2000	0.928	0.937	0.942	0.915	0.915	0.928	0.902	0.904	0.900
	3000	0.947	0.947	0.943	0.936	0.944	0.939	0.909	0.916	0.934
	5000	0.943	0.942	0.940	0.941	0.942	0.934	0.929	0.935	0.920
NORM/IND	200	0.946	0.946	0.943	0.948	0.951	0.933	0.948	0.950	0.945
	500	0.935	0.946	0.954	0.943	0.947	0.955	0.952	0.951	0.959
	800	0.939	0.944	0.947	0.945	0.943	0.948	0.955	0.951	0.924
	1000	0.947	0.933	0.928	0.945	0.952	0.938	0.952	0.940	0.944
	1200	0.951	0.959	0.948	0.960	0.949	0.963	0.959	0.952	0.959
	1500	0.930	0.941	0.947	0.956	0.939	0.952	0.964	0.937	0.949
	1800	0.939	0.951	0.941	0.946	0.946	0.950	0.956	0.955	0.946
	2000	0.953	0.943	0.945	0.950	0.950	0.939	0.943	0.941	0.951
	3000	0.948	0.938	0.953	0.949	0.954	0.957	0.951	0.956	0.941
	5000	0.934	0.944	0.953	0.952	0.965	0.949	0.949	0.951	0.952
T/IND	200	0.918	0.925	0.920	0.911	0.899	0.896	0.842	0.836	0.815
	500	0.926	0.935	0.942	0.898	0.894	0.910	0.836	0.828	0.812
	800	0.930	0.939	0.921	0.899	0.902	0.899	0.831	0.830	0.826
	1000	0.930	0.928	0.941	0.899	0.918	0.908	0.846	0.814	0.850
	1200	0.932	0.933	0.930	0.902	0.894	0.917	0.835	0.845	0.873
	1500	0.941	0.938	0.923	0.922	0.927	0.946	0.867	0.879	0.856
	1800	0.932	0.927	0.941	0.936	0.919	0.928	0.854	0.868	0.880
	2000	0.936	0.927	0.937	0.902	0.909	0.920	0.874	0.878	0.873
	3000	0.942	0.940	0.943	0.931	0.944	0.929	0.898	0.906	0.907
	5000	0.945	0.930	0.941	0.936	0.937	0.931	0.917	0.924	0.903
NORM/AR1	200	0.949	0.949	0.944	0.946	0.949	0.939	0.948	0.952	0.944
	500	0.937	0.948	0.952	0.940	0.941	0.954	0.949	0.952	0.958
	800	0.941	0.942	0.950	0.944	0.942	0.950	0.957	0.953	0.925
	1000	0.944	0.936	0.931	0.943	0.952	0.943	0.955	0.933	0.947
	1200	0.946	0.960	0.948	0.960	0.952	0.958	0.959	0.954	0.954
	1500	0.933	0.938	0.942	0.955	0.941	0.955	0.963	0.938	0.946
	1800	0.936	0.952	0.941	0.945	0.940	0.948	0.956	0.951	0.947
	2000	0.950	0.944	0.949	0.955	0.949	0.939	0.951	0.943	0.951
	3000	0.950	0.933	0.950	0.951	0.952	0.959	0.948	0.955	0.941
	5000	0.939	0.943	0.953	0.952	0.962	0.949	0.951	0.950	0.955
T/AR1	200	0.924	0.934	0.933	0.930	0.928	0.906	0.894	0.883	0.877
	500	0.934	0.942	0.948	0.916	0.920	0.926	0.888	0.869	0.855
	800	0.935	0.952	0.924	0.922	0.921	0.913	0.876	0.872	0.880
	1000	0.935	0.931	0.942	0.922	0.929	0.922	0.883	0.861	0.886
	1200	0.933	0.934	0.931	0.919	0.911	0.925	0.875	0.891	0.901
	1500	0.941	0.943	0.930	0.941	0.937	0.954	0.898	0.903	0.891
	1800	0.928	0.933	0.940	0.947	0.931	0.938	0.889	0.891	0.901
	2000	0.937	0.938	0.936	0.913	0.917	0.929	0.897	0.903	0.892
	3000	0.948	0.944	0.941	0.931	0.947	0.930	0.916	0.919	0.925
	5000	0.946	0.932	0.937	0.940	0.940	0.938	0.931	0.937	0.920

**Table 6:** Empirical coverage probabilities of $\beta _{10}$ based on proposed unconditional variance estimation method with different subset sizes for generalized linear regression model with identity link with α=0.3 for $\hbox {m}^*$=50, $\hbox {m}^\dagger$=200, $\hbox {m}^\wr$ = 500 based on N=1,000 simulations

X/Corr	Size *r*	$\hbox {geom}^*$	$\hbox {beta}^*$	$\hbox {gamma}^*$	$\hbox {geom}^\dagger$	$\hbox {beta}^\dagger$	$\hbox {gamma}^\dagger$	$\hbox {geom}^\wr$	$\hbox {beta}^\wr$	$\hbox {gamma}^\wr$
NORM/CS	200	0.954	0.945	0.940	0.942	0.947	0.948	0.950	0.950	0.947
	500	0.943	0.931	0.937	0.953	0.939	0.944	0.956	0.942	0.946
	800	0.942	0.946	0.943	0.947	0.942	0.936	0.947	0.949	0.942
	1000	0.942	0.950	0.941	0.956	0.949	0.939	0.947	0.942	0.941
	1200	0.944	0.952	0.954	0.941	0.946	0.943	0.943	0.938	0.947
	1500	0.947	0.957	0.941	0.943	0.943	0.937	0.950	0.939	0.945
	1800	0.946	0.943	0.947	0.950	0.948	0.946	0.942	0.945	0.944
	2000	0.948	0.939	0.944	0.945	0.940	0.946	0.941	0.942	0.939
	3000	0.938	0.940	0.938	0.941	0.945	0.949	0.941	0.939	0.942
	5000	0.939	0.946	0.945	0.943	0.939	0.946	0.942	0.941	0.940
T/CS	200	0.938	0.956	0.954	0.962	0.953	0.958	0.952	0.960	0.952
	500	0.947	0.946	0.945	0.949	0.948	0.960	0.959	0.964	0.960
	800	0.951	0.949	0.951	0.969	0.949	0.957	0.959	0.959	0.965
	1000	0.934	0.953	0.954	0.963	0.964	0.953	0.956	0.965	0.963
	1200	0.958	0.952	0.952	0.955	0.963	0.960	0.961	0.960	0.958
	1500	0.949	0.954	0.949	0.952	0.963	0.960	0.958	0.964	0.955
	1800	0.944	0.955	0.951	0.962	0.956	0.961	0.960	0.960	0.956
	2000	0.953	0.944	0.955	0.961	0.958	0.963	0.961	0.960	0.953
	3000	0.955	0.955	0.951	0.958	0.963	0.960	0.958	0.962	0.957
	5000	0.952	0.946	0.956	0.962	0.963	0.959	0.960	0.958	0.960
NORM/IND	200	0.952	0.943	0.933	0.946	0.953	0.945	0.955	0.959	0.951
	500	0.954	0.937	0.951	0.956	0.940	0.944	0.950	0.947	0.954
	800	0.938	0.946	0.948	0.944	0.946	0.943	0.945	0.946	0.948
	1000	0.938	0.946	0.938	0.947	0.948	0.952	0.950	0.953	0.947
	1200	0.945	0.953	0.952	0.944	0.949	0.951	0.948	0.944	0.948
	1500	0.943	0.947	0.948	0.945	0.944	0.946	0.945	0.949	0.948
	1800	0.956	0.952	0.947	0.947	0.944	0.946	0.948	0.946	0.945
	2000	0.936	0.948	0.942	0.946	0.949	0.950	0.947	0.946	0.946
	3000	0.951	0.943	0.940	0.945	0.950	0.946	0.950	0.944	0.945
	5000	0.943	0.949	0.943	0.946	0.948	0.947	0.945	0.948	0.954
T/IND	200	0.941	0.959	0.954	0.958	0.948	0.950	0.951	0.954	0.952
	500	0.948	0.943	0.950	0.957	0.955	0.957	0.955	0.961	0.954
	800	0.952	0.952	0.949	0.957	0.945	0.954	0.952	0.957	0.957
	1000	0.938	0.956	0.958	0.960	0.955	0.952	0.955	0.959	0.953
	1200	0.946	0.948	0.958	0.958	0.959	0.950	0.954	0.958	0.951
	1500	0.950	0.955	0.958	0.950	0.962	0.956	0.951	0.957	0.952
	1800	0.942	0.947	0.948	0.953	0.956	0.954	0.952	0.958	0.951
	2000	0.948	0.948	0.956	0.954	0.951	0.958	0.957	0.956	0.958
	3000	0.956	0.951	0.949	0.951	0.950	0.950	0.953	0.957	0.956
	5000	0.949	0.942	0.953	0.955	0.953	0.952	0.953	0.955	0.955
NORM/AR1	200	0.954	0.949	0.939	0.946	0.949	0.949	0.951	0.954	0.953
	500	0.950	0.938	0.946	0.957	0.940	0.948	0.952	0.947	0.957
	800	0.935	0.947	0.949	0.940	0.947	0.948	0.948	0.945	0.952
	1000	0.941	0.943	0.945	0.951	0.954	0.952	0.951	0.954	0.948
	1200	0.941	0.953	0.945	0.947	0.949	0.953	0.953	0.943	0.950
	1500	0.944	0.951	0.951	0.947	0.945	0.950	0.946	0.950	0.947
	1800	0.956	0.953	0.949	0.945	0.945	0.947	0.947	0.949	0.953
	2000	0.939	0.948	0.946	0.945	0.948	0.950	0.950	0.949	0.951
	3000	0.946	0.944	0.941	0.943	0.951	0.948	0.952	0.948	0.950
	5000	0.946	0.950	0.947	0.945	0.955	0.953	0.951	0.951	0.953
T/AR1	200	0.938	0.956	0.956	0.959	0.950	0.946	0.954	0.955	0.953
	500	0.943	0.947	0.951	0.953	0.954	0.958	0.961	0.961	0.961
	800	0.950	0.951	0.949	0.962	0.950	0.957	0.951	0.964	0.963
	1000	0.936	0.954	0.959	0.964	0.959	0.950	0.957	0.960	0.961
	1200	0.954	0.954	0.956	0.959	0.960	0.954	0.958	0.960	0.961
	1500	0.949	0.959	0.959	0.956	0.962	0.954	0.954	0.959	0.963
	1800	0.942	0.950	0.947	0.959	0.960	0.966	0.962	0.959	0.957
	2000	0.954	0.953	0.959	0.959	0.952	0.958	0.960	0.963	0.963
	3000	0.957	0.947	0.956	0.958	0.959	0.961	0.960	0.963	0.959
	5000	0.947	0.945	0.954	0.960	0.961	0.955	0.960	0.961	0.963

**Table 7:** Empirical coverage probabilities of $\hat{\beta }_{1}$ based on proposed conditional variance estimation method with different subset sizes for generalized linear regression model with logit link and α=0.3 for $\hbox {m}^*$=50, $\hbox {m}^\dagger$=200, $\hbox {m}^\wr$ = 500 based on N=1,000 simulations

X/Corr	Size *r*	$\hbox {geom}^*$	$\hbox {beta}^*$	$\hbox {gamma}^*$	$\hbox {geom}^\dagger$	$\hbox {beta}^\dagger$	$\hbox {gamma}^\dagger$	$\hbox {geom}^\wr$	$\hbox {beta}^\wr$	$\hbox {gamma}^\wr$
NORM/CS	200	0.951	0.937	0.931	0.934	0.923	0.911	0.930	0.908	0.781
	500	0.946	0.945	0.939	0.958	0.935	0.955	0.942	0.943	0.961
	800	0.931	0.943	0.949	0.955	0.939	0.954	0.959	0.953	0.940
	1000	0.932	0.940	0.950	0.949	0.949	0.954	0.950	0.955	0.953
	1200	0.943	0.945	0.944	0.939	0.948	0.941	0.954	0.948	0.950
	1500	0.935	0.949	0.941	0.950	0.958	0.951	0.941	0.954	0.947
	1800	0.948	0.945	0.939	0.960	0.952	0.956	0.959	0.942	0.947
	2000	0.956	0.949	0.940	0.956	0.944	0.937	0.956	0.943	0.952
	3000	0.947	0.947	0.944	0.949	0.944	0.955	0.950	0.950	0.947
	5000	0.958	0.951	0.954	0.947	0.947	0.947	0.946	0.948	0.954
NORM/IND	200	0.941	0.946	0.934	0.941	0.941	0.931	0.928	0.939	0.925
	500	0.945	0.932	0.938	0.956	0.937	0.946	0.949	0.942	0.952
	800	0.936	0.936	0.944	0.954	0.951	0.953	0.947	0.942	0.936
	1000	0.937	0.946	0.940	0.943	0.943	0.957	0.942	0.956	0.954
	1200	0.943	0.944	0.940	0.942	0.949	0.938	0.961	0.943	0.944
	1500	0.950	0.939	0.955	0.946	0.956	0.946	0.940	0.963	0.944
	1800	0.946	0.938	0.943	0.958	0.953	0.950	0.952	0.945	0.955
	2000	0.957	0.932	0.947	0.952	0.951	0.947	0.954	0.954	0.956
	3000	0.942	0.942	0.946	0.958	0.945	0.946	0.934	0.950	0.944
	5000	0.949	0.952	0.957	0.946	0.953	0.940	0.957	0.954	0.958
NORM/AR1	200	0.962	0.940	0.947	0.939	0.936	0.911	0.929	0.915	0.834
	500	0.956	0.947	0.935	0.948	0.943	0.956	0.941	0.945	0.957
	800	0.931	0.945	0.944	0.949	0.949	0.954	0.946	0.940	0.946
	1000	0.936	0.937	0.952	0.947	0.943	0.951	0.944	0.961	0.947
	1200	0.945	0.941	0.942	0.942	0.945	0.944	0.959	0.943	0.957
	1500	0.946	0.939	0.939	0.950	0.952	0.957	0.951	0.943	0.952
	1800	0.945	0.948	0.944	0.955	0.958	0.954	0.964	0.950	0.953
	2000	0.950	0.948	0.932	0.954	0.959	0.941	0.954	0.953	0.949
	3000	0.950	0.953	0.941	0.958	0.940	0.949	0.947	0.946	0.953
	5000	0.953	0.948	0.956	0.953	0.944	0.938	0.948	0.947	0.960

**Table 8:** Empirical coverage probabilities of $\beta _{10}$ based on proposed unconditional variance estimation method with different subset sizes for generalized linear regression model with logit link and α=0.3 for $\hbox {m}^*$=50, $\hbox {m}^\dagger$=200, $\hbox {m}^\wr$ = 500 based on N=1,000 simulations

X/Corr	Size *r*	$\hbox {geom}^*$	$\hbox {beta}^*$	$\hbox {gamma}^*$	$\hbox {geom}^\dagger$	$\hbox {beta}^\dagger$	$\hbox {gamma}^\dagger$	$\hbox {geom}^\wr$	$\hbox {beta}^\wr$	$\hbox {gamma}^\wr$
NORM/CS	200	0.943	0.942	0.938	0.952	0.950	0.946	0.958	0.947	0.938
	500	0.949	0.953	0.949	0.948	0.953	0.963	0.954	0.961	0.961
	800	0.932	0.941	0.962	0.953	0.956	0.955	0.954	0.958	0.957
	1000	0.949	0.949	0.949	0.959	0.956	0.963	0.955	0.963	0.955
	1200	0.958	0.945	0.953	0.959	0.956	0.953	0.960	0.956	0.959
	1500	0.947	0.950	0.952	0.954	0.955	0.959	0.959	0.960	0.954
	1800	0.949	0.950	0.954	0.958	0.954	0.958	0.952	0.960	0.957
	2000	0.942	0.952	0.947	0.953	0.958	0.958	0.955	0.955	0.956
	3000	0.953	0.952	0.954	0.949	0.959	0.958	0.954	0.957	0.957
	5000	0.952	0.948	0.956	0.959	0.943	0.957	0.961	0.959	0.957
NORM/IND	200	0.947	0.953	0.951	0.953	0.959	0.942	0.950	0.959	0.957
	500	0.949	0.952	0.940	0.949	0.954	0.950	0.949	0.959	0.952
	800	0.935	0.951	0.956	0.946	0.948	0.955	0.951	0.953	0.951
	1000	0.949	0.942	0.947	0.953	0.959	0.954	0.954	0.951	0.954
	1200	0.949	0.944	0.942	0.949	0.948	0.955	0.954	0.953	0.950
	1500	0.958	0.951	0.950	0.952	0.951	0.955	0.958	0.956	0.953
	1800	0.952	0.946	0.946	0.952	0.949	0.956	0.952	0.956	0.955
	2000	0.953	0.948	0.951	0.947	0.959	0.952	0.954	0.951	0.954
	3000	0.946	0.950	0.955	0.948	0.951	0.954	0.951	0.956	0.952
	5000	0.943	0.946	0.947	0.954	0.954	0.955	0.951	0.955	0.955
NORM/AR1	200	0.942	0.946	0.940	0.948	0.954	0.942	0.948	0.949	0.945
	500	0.952	0.953	0.948	0.949	0.955	0.956	0.958	0.960	0.954
	800	0.940	0.946	0.950	0.953	0.955	0.956	0.953	0.956	0.954
	1000	0.943	0.948	0.956	0.954	0.952	0.960	0.955	0.954	0.957
	1200	0.958	0.951	0.954	0.955	0.952	0.955	0.952	0.959	0.958
	1500	0.946	0.956	0.953	0.954	0.956	0.956	0.953	0.958	0.959
	1800	0.939	0.943	0.945	0.955	0.954	0.952	0.954	0.955	0.961
	2000	0.946	0.945	0.948	0.954	0.959	0.953	0.955	0.955	0.955
	3000	0.952	0.951	0.950	0.951	0.958	0.956	0.954	0.959	0.955
	5000	0.953	0.953	0.949	0.953	0.949	0.959	0.956	0.955	0.956

**Table 9:** Empirical conditional MSE of proposed repeated perturbation estimator with m=20 compared to other methods with different subset sizes for SHMAS dataset using generalized linear regression model with natural link and logit link. The empirical MSE are the averages obtained based on N=1,000 different subsets from the full dataset

	Size	unisMUL	unisGEOM	geomPERT	betaPERT	gammaPERT
Sleepiness check	100	1.701	1.635	0.139	0.102	0.082
	200	0.802	0.681	0.066	0.050	0.040
	300	0.556	0.431	0.041	0.030	0.026
	500	0.311	0.203	0.020	0.015	0.015
	800	0.184	0.085	0.008	0.008	0.010
	1000	0.145	0.044	0.004	0.005	0.008
Sleep quality	100	2.963	2.790	0.289	0.210	0.147
	200	1.182	1.034	0.107	0.077	0.064
	300	0.756	0.564	0.060	0.044	0.038
	500	0.431	0.277	0.027	0.021	0.022
	800	0.257	0.104	0.010	0.010	0.012
	1000	0.204	0.050	0.005	0.006	0.011

**Table 10:** Coverage rate of $\hat{\beta }_{Gender}$ and $\hat{\beta }_{Good life}$ based on the proposed repeated perturbation variance estimator with m=20 with different subset sizes for SHMAS dataset using generalized linear regression model with natural link and logit link. The coverage rate are the averages obtained based on N=1,000 different subsets from the full dataset

		Sleepiness check			Sleep quality		
	Size	geomPERT	betaPERT	gammaPERT	geomPERT	betaPERT	gammaPERT
$\hat{\beta }_{Gender}$	100	0.931	0.934	0.936	0.922	0.934	0.944
	200	0.942	0.935	0.934	0.936	0.932	0.938
	300	0.943	0.948	0.951	0.941	0.939	0.947
	500	0.941	0.935	0.938	0.933	0.935	0.941
	800	0.927	0.938	0.937	0.924	0.930	0.945
	1000	0.938	0.940	0.926	0.928	0.941	0.948
$\hat{\beta }_{Good life}$	100	0.931	0.934	0.945	0.928	0.940	0.910
	200	0.927	0.914	0.928	0.934	0.914	0.922
	300	0.933	0.944	0.946	0.931	0.918	0.928
	500	0.942	0.944	0.941	0.929	0.924	0.919
	800	0.923	0.940	0.940	0.929	0.928	0.934
	1000	0.939	0.942	0.926	0.940	0.938	0.939
